# Supplementary material for: The Association between Baseline Proton Pump Inhibitors, Immune Checkpoint Inhibitors, and Chemotherapy: A Systematic Review with Network Meta-Analysis
Source: Cancers (Basel). 2022 Dec 31;15(1):284. doi: 10.3390/cancers15010284 (PMC9818995; doi:10.3390/cancers15010284)
Supplement: Supplementary file 1 [file cancers-15-00284-s001.zip › cancers-2112483-supplementary.pdf]

Supplementary file

Contents of supplements

Method 1. PRISMA NMA Checklist of Items to Include When Reporting A Systematic Review Involving a Network Meta-analysis. PRISMA, Preferred Reporting Items for Systematic Reviews and Meta-Analyses; NMA, network meta-analysis

Method 2. Preferred Reporting Items for Systematic Reviews and Meta-Analyses (PRISMA) 2020 checklist

Method 3. Meta-analysis Of Observational Studies in Epidemiology (MOOSE) checklist

Method 4. Search strategy

Method 5. Data extraction and data synthesis

Result 1. Details of full-text inspection

Result 2. Details of risk of bias assessment for survival outcome *(Figure S1)*

Result 3. Details of NMA of overall survival and progression-free survival *(Figure S2–7)*

Result 4. Network meta-analysis after excluding melanoma trials *(Figure S8–16)*

Result 5. Sensitivity analyses of NMA, excluding 1st line therapy *(Figure S17–23)*

Result 6. Sensitivity analyses of NMA, excluding unknown PDL-1 status *(Figure S24–30)*

Result 7. Assessment of publication bias in pairwise meta-analysis of ICI cohorts *(Figure S31–32)*

Result 8. Sensitivity analysis of excluding studies of high risk of bias *(Figure S33–34)*

Result 9. Pairwise meta-analyses of chemotherapy cohorts *(Figure S35–38)*

Result 10. Pairwise meta-analyses using adjusted Hazard Ratios (HR) (*Figure S39–44*)

Table S1. Eligibility criteria of trial patients included in NMA

Table S2. Details of uncategorized cancers

Table S3. Effect modifiers across the network

Table S4. Details of proton pump inhibitors

Table S5. Covariates of studies reporting adjusted estimates

Table S6. Summary of findings of our meta-analysis

This supplementary material has been provided by the authors to give readers additional information about their work.

Method 1. PRISMA NMA Checklist of Items to Include When Reporting A Systematic Review Involving a Network Meta-analysis

Section/Topic	Item #	Checklist Item	Reported on Page #
**TITLE**			
Title	1	Identify the report as a systematic review *incorporating a network meta-analysis (or related form of meta-analysis).*	# 1
**ABSTRACT**			
Structured summary	2	Provide a structured summary including, as applicable:<br>**Background:** main objectives<br>**Methods:** data sources; study eligibility criteria, participants, and interventions; study appraisal; and *synthesis methods, such as network meta-analysis.*<br>**Results:** number of studies and participants identified; summary estimates with corresponding confidence/credible intervals; *treatment rankings may also be discussed. Authors may choose to summarize pairwise comparisons against a chosen treatment included in their analyses for brevity.*<br>**Discussion/Conclusions:** limitations; conclusions and implications of findings.<br>**Other:** primary source of funding; systematic review registration number with registry name.	# 5
**INTRODUCTION**			
Rationale	3	Describe the rationale for the review in the context of what is already known*, including mention of why a network meta-analysis has been conducted.*	# 7
Objectives	4	Provide an explicit statement of questions being addressed, with reference to participants, interventions, comparisons, outcomes, and study design (PICOS).	# 7
**METHODS**			
Protocol and registration	5	Indicate whether a review protocol exists and if and where it can be accessed (e.g., Web address); and, if available, provide registration information, including registration number.	# 8
Eligibility criteria	6	Specify study characteristics (e.g., PICOS, length of follow-up) and report characteristics (e.g., years considered, language, publication status) used as criteria for eligibility, giving rationale. *Clearly describe eligible treatments included in the treatment network, and note whether any have been clustered or merged into the same node (with justification).*	# 10
Information sources	7	Describe all information sources (e.g., databases with dates of coverage, contact with study authors to identify additional studies) in the search and date last searched.	# 8
Search	8	Present full electronic search strategy for at least one database, including any limits used, such that it could be	Method 4

		repeated.	
Study selection	9	State the process for selecting studies (i.e., screening, eligibility, included in systematic review, and, if applicable, included in the meta-analysis).	# 8
Data collection process	10	Describe method of data extraction from reports (e.g., piloted forms, independently, in duplicate) and any processes for obtaining and confirming data from investigators.	Method 5
Data items	11	List and define all variables for which data were sought (e.g., PICOS, funding sources) and any assumptions and simplifications made.	# 9
**Geometry of the network**	**S1**	Describe methods used to explore the geometry of the treatment network under study and potential biases related to it. This should include how the evidence base has been graphically summarized for presentation, and what characteristics were compiled and used to describe the evidence base to readers.	# 10
Risk of bias within individual studies	12	Describe methods used for assessing risk of bias of individual studies (including specification of whether this was done at the study or outcome level), and how this information is to be used in any data synthesis.	# 9
Summary measures	13	State the principal summary measures (e.g., risk ratio, difference in means). *Also describe the use of additional summary measures assessed, such as treatment rankings and surface under the cumulative ranking curve (SUCRA) values, as well as modified approaches used to present summary findings from meta-analyses.*	# 9
Planned methods of analysis	14	Describe the methods of handling data and combining results of studies for each network meta-analysis. This should include, but not be limited to:<br>● *Handling of multi-arm trials;*<br>● *Selection of variance structure;*<br>● *Selection of prior distributions in Bayesian analyses; and*<br>● *Assessment of model fit.*	# 10
**Assessment of Inconsistency**	**S2**	Describe the statistical methods used to evaluate the agreement of direct and indirect evidence in the treatment network(s) studied. Describe efforts taken to address its presence when found.	# 10
Risk of bias across studies	15	Specify any assessment of risk of bias that may affect the cumulative evidence (e.g., publication bias, selective reporting within studies).	# 10
Additional analyses	16	Describe methods of additional analyses if done, indicating which were pre-specified. This may include, but not be limited to, the following:<br>● Sensitivity or subgroup analyses;<br>● Meta-regression analyses;<br>● *Alternative formulations of the treatment network; and*<br>● *Use of alternative prior distributions for Bayesian analyses (if applicable).*	# 11

**RESULTS†**			
Study selection	17	Give numbers of studies screened, assessed for eligibility, and included in the review, with reasons for exclusions at each stage, ideally with a flow diagram.	Figure S1
**Presentation of network structure**	**S3**	Provide a network graph of the included studies to enable visualization of the geometry of the treatment network.	Figure S2
**Summary of network geometry**	**S4**	Provide a brief overview of characteristics of the treatment network. This may include commentary on the abundance of trials and randomized patients for the different interventions and pairwise comparisons in the network, gaps of evidence in the treatment network, and potential biases reflected by the network structure.	# 13
Study characteristics	18	For each study, present characteristics for which data were extracted (e.g., study size, PICOS, follow-up period) and provide the citations.	Table S1
Risk of bias within studies	19	Present data on risk of bias of each study and, if available, any outcome level assessment.	Result 2
Results of individual studies	20	For all outcomes considered (benefits or harms), present, for each study: 1) simple summary data for each intervention group, and 2) effect estimates and confidence intervals. *Modified approaches may be needed to deal with information from larger networks.*	Result 3
Synthesis of results	21	Present results of each meta-analysis done, including confidence/credible intervals. *In larger networks, authors may focus on comparisons versus a particular comparator (e.g. placebo or standard care), with full findings presented in an appendix. League tables and forest plots may be considered to summarize pairwise comparisons.* If additional summary measures were explored (such as treatment rankings), these should also be presented.	Result 3
**Exploration for inconsistency**	**S5**	Describe results from investigations of inconsistency. This may include such information as measures of model fit to compare consistency and inconsistency models, *P* values from statistical tests, or summary of inconsistency estimates from different parts of the treatment network.	Result 3
Risk of bias across studies	22	Present results of any assessment of risk of bias across studies for the evidence base being studied.	Figure S8, S9
Results of additional analyses	23	Give results of additional analyses, if done (e.g., sensitivity or subgroup analyses, meta-regression analyses*, alternative network geometries studied, alternative choice of prior distributions for Bayesian analyses,* and so forth).	# 13, 14
**DISCUSSION**			
Summary of evidence	24	Summarize the main findings, including the strength of evidence for each main outcome; consider their relevance to key groups (e.g., healthcare providers, users, and policy-makers).	# 16
Limitations	25	Discuss limitations at study and outcome level (e.g., risk of bias), and at review level (e.g., incomplete retrieval of identified research, reporting bias). *Comment on the validity*	# 22

		of the assumptions, such as transitivity and consistency. Comment on any concerns regarding network geometry (e.g., avoidance of certain comparisons).	
Conclusions	26	Provide a general interpretation of the results in the context of other evidence, and implications for future research.	# 22
**FUNDING**			N/A
Funding	27	Describe sources of funding for the systematic review and other support (e.g., supply of data); role of funders for the systematic review. This should also include information regarding whether funding has been received from manufacturers of treatments in the network and/or whether some of the authors are content experts with professional conflicts of interest that could affect use of treatments in the network.	

Method 2. PRISMA 2020 checklist

Section and Topic	Item #	Checklist item	Location where item is reported
**TITLE**			
Title	1	Identify the report as a systematic review.	Page 1
**ABSTRACT**			
Abstract	2	See the PRISMA 2020 for Abstracts checklist.	Page 5
**INTRODUCTION**			
Rationale	3	Describe the rationale for the review in the context of existing knowledge.	Page 7
Objectives	4	Provide an explicit statement of the objective(s) or question(s) the review addresses.	Page 7
**METHODS**			
Eligibility criteria	5	Specify the inclusion and exclusion criteria for the review and how studies were grouped for the syntheses.	Page 8
Information sources	6	Specify all databases, registers, websites, organisations, reference lists and other sources searched or consulted to identify studies. Specify the date when each source was last searched or consulted.	Page 8
Search strategy	7	Present the full search strategies for all databases, registers and websites, including any filters and limits used.	eMetohd 4
Selection process	8	Specify the methods used to decide whether a study met the inclusion criteria of the review, including how many reviewers screened each record and each report retrieved, whether they worked independently, and if applicable, details of automation tools used in the process.	Page 8
Data collection process	9	Specify the methods used to collect data from reports, including how many reviewers collected data from each report, whether they worked independently, any processes for obtaining or confirming data from study investigators, and if applicable, details of automation tools used in the process.	Method 5
Data items	10a	List and define all outcomes for which data were sought. Specify whether all results that were compatible with each outcome domain in each study were sought (e.g. for all measures, time points, analyses), and if not, the methods used to decide which results to collect.	Page 9
	10b	List and define all other variables for which data were sought (e.g. participant and intervention characteristics, funding sources). Describe any assumptions made about any missing or unclear information.	Method 5
Study risk of bias assessment	11	Specify the methods used to assess risk of bias in the included studies, including details of the tool(s) used, how many reviewers assessed each study and whether they worked independently, and if applicable, details of automation tools used in the process.	Page 9
Effect measures	12	Specify for each outcome the effect measure(s) (e.g. risk ratio, mean difference) used in the synthesis or presentation of results.	Page 9
Synthesis methods	13a	Describe the processes used to decide which studies were eligible for each synthesis (e.g. tabulating the study intervention characteristics and comparing against the planned groups for each synthesis (item #5)).	Method 5
	13b	Describe any methods required to prepare the data for presentation or synthesis, such as handling of missing summary statistics, or data conversions.	Method 5
	13c	Describe any methods used to tabulate or visually display results of individual studies and syntheses.	Method 5
	13d	Describe any methods used to synthesize results and provide a rationale for the choice(s). If meta-analysis was performed, describe the	Method 5

		model(s), method(s) to identify the presence and extent of statistical heterogeneity, and software package(s) used.	
	13e	Describe any methods used to explore possible causes of heterogeneity among study results (e.g. subgroup analysis, meta-regression).	Page 9, 10
	13f	Describe any sensitivity analyses conducted to assess robustness of the synthesized results.	Page 9, 10
Reporting bias assessment	14	Describe any methods used to assess risk of bias due to missing results in a synthesis (arising from reporting biases).	Page 10
Certainty assessment	15	Describe any methods used to assess certainty (or confidence) in the body of evidence for an outcome.	N/A
**RESULTS**			
Study selection	16a	Describe the results of the search and selection process, from the number of records identified in the search to the number of studies included in the review, ideally using a flow diagram.	Figure S1
	16b	Cite studies that might appear to meet the inclusion criteria, but which were excluded, and explain why they were excluded.	Result 1
Study characteristics	17	Cite each included study and present its characteristics.	Table S1
Risk of bias in studies	18	Present assessments of risk of bias for each included study.	Figure S1
Results of individual studies	19	For all outcomes, present, for each study: (a) summary statistics for each group (where appropriate) and (b) an effect estimate and its precision (e.g. confidence/credible interval), ideally using structured tables or plots.	Result 3
Results of syntheses	20a	For each synthesis, briefly summarize the characteristics and risk of bias among contributing studies.	Results 2
	20b	Present results of all statistical syntheses conducted. If meta-analysis was done, present for each the summary estimate and its precision (e.g. confidence/credible interval) and measures of statistical heterogeneity. If comparing groups, describe the direction of the effect.	Page 14, 15
	20c	Present results of all investigations of possible causes of heterogeneity among study results.	Page 14, 15
	20d	Present results of all sensitivity analyses conducted to assess the robustness of the synthesized results.	Page 14, 15
Reporting biases	21	Present assessments of risk of bias due to missing results (arising from reporting biases) for each synthesis assessed.	Result 7
Certainty of evidence	22	Present assessments of certainty (or confidence) in the body of evidence for each outcome assessed.	N/A
**DISCUSSION**			
Discussion	23a	Provide a general interpretation of the results in the context of other evidence.	Page 16
	23b	Discuss any limitations of the evidence included in the review.	Page 22
	23c	Discuss any limitations of the review processes used.	Page 22
	23d	Discuss implications of the results for practice, policy, and future research.	Page 22
**OTHER INFORMATION**			
Registration and	24a	Provide registration information for the review, including register name and registration number, or state that the review was not registered.	

protocol	24b	Indicate where the review protocol can be accessed, or state that a protocol was not prepared.	Page 8
	24c	Describe and explain any amendments to information provided at registration or in the protocol.	Page 8
Support	25	Describe sources of financial or non-financial support for the review, and the role of the funders or sponsors in the review.	N/A
Competing interests	26	Declare any competing interests of review authors.	N/A
Availability of data, code and other materials	27	Report which of the following are publicly available and where they can be found: template data collection forms; data extracted from included studies; data used for all analyses; analytic code; any other materials used in the review.	Method 5

Method 3. MOOSE checklist

Reporting Criteria	Reported (Yes/No)	Reported on Page No.	Brief description
**Reporting of Background**			
Problem definition	Yes	7	The interaction of PPI with ICI versus chemotherapy in cancer patients has not been clarified.
Hypothesis statement	Yes	8	The comparative survival outcomes of patients treated with ICI and chemotherapy would be influenced by the use of PPI.
Description of Study Outcome(s)	Yes	8	Survival outcomes of cancer patients treated with ICI or chemotherapy.
Type of exposure or intervention used	Yes	8	Cancer patients with and without baseline PPI use
Type of study design used	Yes	8	Randomized controlled trials, Prospective and retrospective cohort studies
Study population	Yes	8	Cancer patients treated with ICI or chemotherapy.
**Reporting of Search Strategy**			
Qualifications of searchers (eg, librarians and investigators)	Yes	7	W.Y.L, Y.C, and Y.C.C
Search strategy, including time period included in the synthesis and keywords	Yes	Method 4	As shown in Method 4
Effort to include all available studies, including contact with authors	Yes	N/A	We searched bibliographies of retrieved references.<br>It is unnecessary to contact authors as the data were publicly available.
Databases and registries searched	Yes	Method 4	PubMed, Embase, Cochrane librar, and Medline
Search software used, name and version, including special features used (eg, explosion)	Yes	Method 4	Endnote X 9.3 was used to manage reference
Use of hand searching (eg, reference lists of obtained articles)	Yes	Method 4	We searched bibliographies of retrieved references.
List of citations located and those excluded, including justification	Yes	Result 1	As shown in eResult 1
Method for addressing articles published in languages other than English	Yes	N/A	We used translation software for addressing articles published in languages.
Method of handling abstracts and unpublished studies	Yes	8, 9	We included conference abstract.
Description of any contact with authors	Yes	N/A	It is unnecessary to contact authors as the data were publicly available.

**Reporting of Methods**			
Description of relevance or appropriateness of studies assembled for assessing the hypothesis to be tested	Yes	8	(1) randomized controlled trials, prospective or retrospective cohort studies;<br>(2) studies involving adult patients aged over 18 with cancers receiving ICI or chemotherapy;<br>(3) studies reporting at least one comparative survival outcome
Rationale for the selection and coding of data (eg, sound clinical principles or convenience)	Yes	8	Three investigators (K.Y.C, Y.C, and W.Y.L) independently extracted relevant information from eligible articles.
Documentation of how data were classified and coded (eg, multiple raters, blinding, and interrater reliability)	Yes	Method 5	Details of extraction are in Method 5.
Assessment of confounding (eg, comparability of cases and controls in studies where appropriate	Yes	9	Three reviewers (K.Y.C, Y.C and W.Y.L) independently completed a critical appraisal of included literature by using the Risk Of Bias In Non-randomized Studies - of Interventions (ROBINS-I) tool for each clinical outcome.
**Reporting Criteria**			
Assessment of study quality, including blinding of quality assessors; stratification or regression on possible predictors of study results	Yes	9	ROBINS-I was applied.
Assessment of heterogeneity	Yes	11	Heterogeneity was assessed using I^2 statistics proposed by Higgins and Thompson.
Description of statistical methods (eg, complete description of fixed or random effects models, justification of whether the chosen models account for predictors of study results, dose-response models, or cumulative meta-analysis) in sufficient detail to be replicated	Yes	Method 5	We performed meta-analysis using random-effects model and meta-regression was conducted using a random-effects model. RStudio's ''metafor'' package was used for all analyses.
Provision of appropriate tables and graphics	Yes	Figures and Tables	We included tables for illustrating details of included the studies and figures demonstrating a flow chart of study identification and the results of the meta-analyses.
**Reporting of Results**			
Table giving descriptive information for each study included	Yes	Table S1	Details are in Table S1

Results of sensitivity testing (eg, subgroup analysis)	Yes	14, 15	We performed subgroup analysis based on different treatment modalities and cancer types.
Indication of statistical uncertainty of findings	Yes	14, 15	95% confidence intervals and I^2 values were presented with all effect estimates
**Reporting of Discussion**			
Quantitative assessment of bias (eg, publication bias)	Yes	Result 7	As shown in Result 7
Justification for exclusion (eg, exclusion of non–English-language citations)	Yes	N/A	Studies were excluded based on the pre-specified eligibility criteria in Method.
Assessment of quality of included studies	Yes	Result 1	As shown in Result 1
Reporting of Conclusions			
Consideration of alternative explanations for observed results	Yes	16-21	Elaborated in Discussion
Generalization of the conclusions (ie, appropriate for the data presented and within the domain of the literature review)	Yes	22	The prognostic effects of PPI-induced dysbiosis on ICI-containing regimens are dependent on cancer types. In certain cancers.
Guidelines for future research	Yes	22	Future RCTs of high quality including: more cancer types, and more detailed PDL-1 status are warranted to elucidate their effects on immunotherapy and chemotherapy.
Disclosure of funding source	Yes	N/A	N/A

Method 4. Search strategy

PubMed (12060)
((PPI) or (proton pump inhibitor) or (gastric acid suppressants) or (H2 blocker) or (H2 antagonist) or (H2 receptor antagonist) or (esomeprazole) or (omeprazole) or (pantoprazole) or (rabeprazole) or (lansoprazole) or (dexlansoprazole) or (cimetidine) or (ranitidine) or (famotidine) or (nizatidine) or (comedication) or (concomitant medication)) and ((Immunotherapy) or (immune therapy) or (immune check point inhibitor) or (PD1) or (PDL1) or (CTLA4) or (programmed cell death protein 1) or (programmed death ligand 1) or (cytotoxic t lymphocyte associated Protein 4) or (durvalumab) or (avelumab) or (atezolizumab) or (pembrolizumab) or (lambrolizumab) or (nivolumab) or (Ipilimumab) or (tremelimumab) or (ticilimumab) or (chemotherapeutic agent) or (chemotherapy)) and ((cancer) or (carcinoma) or (solid tumor) or (malignancy) or (melanoma) or (malignant neoplasm))

Embase

Search Number	Search Description	Numbers of results
1	(((PPI) or (proton pump inhibitor) or (gastric acid suppressants) or (H2 blocker) or (H2 antagonist) or (H2 receptor antagonist))):ti,ab,kw,de	87562
2	(esomeprazole or omeprazole or pantoprazole or rabeprazole or lansoprazole orlanzoprazole or dexlansoprazole or cimetidine or ranitidine or famotidine or nizatidine or comedication or concomitant medication):ti,ab,kw,de	74447
3	'proton pump inhibitor'/exp	89465
4	'histamine H2 receptor antagonist'/exp	75759
5	((Immunotherapy) or (immune therapy) or (immune check point inhibitor) or (PD1) or (PDL1) or (CTLA4) or (programmed cell death protein 1) or (programmed death ligand 1) or (cytotoxic t lymphocyte associated Protein 4) or durvalumab or avelumab or atezolizumab or pembrolizumab or lambrolizumab or nivolumab or Ipilimumab or tremelimumab or ticilimumab):ti,ab,kw,de	552080
6	((chemotherapeutic agent) or chemotherapy):ti,ab,kw,de	952506
7	'immunotherapy'/exp	269095
8	'chemotherapy'/exp	760094
9	((cancer) or (carcinoma) or (solid tumor) or (malignan*) or (melanoma)):ti,ab,kw,de	4951303
10	'malignant neoplasm'/exp	4207460
11	(#1 or #2 or #3 or #4) and (#5 or #6 or #7 or #8) and (#9 or #10)	7424

Medline

Search Number	Search Description	Numbers of results
1	(((PPI) or (proton pump inhibitor) or (gastric acid suppressants) or (H2 blocker) or (H2 antagonist) or (H2 receptor antagonist))).mp	31851
2	(esomeprazole or omeprazole or pantoprazole or rabeprazole or lansoprazole orlanzoprazole or dexlansoprazole or cimetidine or ranitidine or famotidine or nizatidine or comedication or (concomitant medication)).mp	35443
3	exp Proton Pump Inhibitors/	200577
4	exp Histamine H2 Antagonists/	19391
5	((Immunotherapy) or (immune therapy) or (immune check point inhibitor) or (PD1) or (PDL1) or (CTLA4) or (programmed cell death protein 1) or (programmed death ligand 1) or (cytotoxic t lymphocyte associated Protein 4) or<br>durvalumab or avelumab or atezolizumab or pembrolizumab or lambrolizumab or nivolumab or Ipilimumab or tremelimumab or ticilimumab).mp	158549
6	((chemotherapeutic agent) or chemotherapy).mp	506269
7	exp immunotherapy/	313477
8	((cancer) or (carcinoma) or (solid tumor) or (malignan*) or (melanoma)).mp	2930136
9	Exp Neoplasms/	3687792
10	(#1 or #2 or #3 or #4) and (#5 or #6 or #7) and (#8 or #9)	932

Cochrane library

#1	(((PPI) or (proton pump inhibitor) or (gastric acid suppressants) or (H2 blocker) or (H2 antagonist) or (H2 receptor antagonist)))	Limits	5389
#2	(esomeprazole or omeprazole or pantoprazole or rabeprazole or lansoprazole orlanzoprazole or dexlansoprazole or cimetidine or ranitidine or famotidine or nizatidine or comedication or (concomitant medication))	Limits	17226
#3	MeSH descriptor: [Proton Pump Inhibitors] explode all trees	MeSH	1551
#4	MeSH descriptor: [Histamine H2 Antagonists] explode all trees	MeSH	890
#5	((Immunotherapy) or (immune therapy) or (immune check point inhibitor) or (PD1) or (PDL1) or (CTLA4) or (programmed cell death protein 1) or (programmed death ligand 1) or (cytotoxic t lymphocyte associated Protein 4) or<br>durvalumab or avelumab or atezolizumab or pembrolizumab or lambrolizumab or nivolumab or Ipilimumab or tremelimumab or ticilimumab)	Limits	35619
#6	((chemotherapeutic agent) or chemotherapy)	Limits	89215
#7	MeSH descriptor: [Immunotherapy] explode all trees	MeSH	8657
#8	((cancer) or (carcinoma) or (solid tumor) or (malignan*) or (melanoma))	Limits	224985
#9	MeSH descriptor: [Neoplasms] explode all trees	MeSH	87970
#10	(#1 or #2 or #3 or #4) and (#5 or #6 or #7) and (#8 or #9)	Limits	643

Method 5. Data extraction and data synthesis

Three investigators (K.Y.C, Y.C, and W.Y.L) independently extracted relevant information from eligible articles, including (1) first author's name with publication year, (2) study type, (3) country, (4) inclusion period, (5) sample size, (6) cancer type, (7) therapeutic modality, (8) first line proportion, (9) PPI users, (10) PPI using window, (11) different types of PPI, (12) duration of follow up, and (13) patient's demographics: age, sex [male percentage], ECOG-PS of 0, 1, 2, and PDL-1 expression status of IC0, and IC1/2/3. For studies enrolling mixed type of cancers or ICI, we attempt to break down the proportion of individual cancer and ICI.

We used Rstudio with meta, metafor, and netmeta packages to conduct statistical analysis:

【meta】package
Random-effects model:
Meta-analysis of binary outcome data (metabin)
Meta-analysis of continuous outcome data (metacont)
Generic inverse variance meta-analysis (metagen)
Several plots for meta-analysis: Forest plot (forest)
Funnel plot (funnel)
Exploring biases:
Egger's test (metabias)

【metafor】packages
Meta-regression (metareg)

【netmeta】packages
Network meta-analysis (netmeta)
Network plot (netgraph)
Netheat plot (netheat)
Split direct and indirect evidence (netsplit)
League table (netleague)
Comparison-adjusted funnel plot (funnel)

```
#Network meta-analysis
PPInet<-read_excel('/Users/kychi/Desktop/ICI and PPI/NMA/PPINMA.xlsx', sheet = 'OS')
PPI.netmeta1 <- netmeta(TE = TE,seTE = seTE,treat1 = treat1,treat2 = treat2,studlab = study,
                data = PPInet,sm = "HR",comb.fixed = F,comb.random = T,
                reference.group = 'Chemotherapy', details.chkmultiarm = T,sep.trts = " versus ")
PPI.netmeta1
netgraph(PPI.netmeta1, points = T, cex.points =6 , cex = 1, thickness = F,
           plastic = F, col.points = ‘red’, col = ‘blue’, iterate = T)
netheat (PPI.netmeta1, random = T)
netsplit1<-netsplit(PPI.netmeta)
netsplit1
forest(netsplit1, fontsize = 10, spacing = 0.8,
     col.diamond = 'red', col.diamond.lines ='red',
     ff.xlab="bold", col.by='black', col.square = 'blue', col.square.lines = 'blue')
netleague <- netleague(PPI.netmeta,
                 bracket = "(", digits=2, separator='-', seq= netrank(PPI.netmeta))
netleague
ord <- c( “Chemo&PPI”, “ICI”, “ICI&PPI”,”Chemotherapy”)
funnel(PPI.netmeta1, order = ord,  col = 1:3, legend = F,
     linreg = TRUE, digits.pval = 2)
f1 <- funnel(PPI.netmeta1, order = ord,  col = 1:3,
          linreg = TRUE, digits.pval = 2)
metabias(metagen(TE.adj, seTE, data = f1))
metabias(metagen(TE.adj, seTE, data = f1), method = “rank”)
metabias(metagen(TE.adj, seTE, data = f1), method = “mm”)
forest(PPI.netmeta1, reference.group = "Chemotherapy", sortvar = TE,
       smlab = paste("Other  therapeutic combinations vs. Chemotherapy alone\n",
       "(Overall  Survival)"), reference.group = TRUE,
       label.left = "Favors other therapeutic combinations",
       label.right = "Favors Chemotherapy alone")
```

```
#Pairwise meta-analysis
un1<-read_xlsx('/Users/kychi/Desktop/ICI and PPI/Pairwise/Final analysis_unadjusted.xlsx',
sheet = 'ICI_OS')
ICI_OS<-metagen(TE, seTE, studlab = study, data = un1, comb.fixed = F,
          sm = 'HR', method.tau = 'REML', byvar = cancer)
forest(ICI_OS, layout = 'RevMan5', lab.e="With PPI", lab.c="Without PPI",
    xlab="Favors PPI users   Favors non-PPI users", ff.xlab="bold",
    col.by="black", comb.fixed=F, col.diamond.random='red',
    col.diamond.lines.random='red', col.square = 'blue', col.square.lines = 'blue',
    print.byvar = F, test.subgroup.random = T, fontsize = 8, spacing = 0.75)

ICI_OS2<-metagen(TE, seTE, studlab = study, data = un1, comb.fixed = F,
          sm = 'HR', method.tau = 'REML')
funnel (ICI_OS2, comb.random = F, contour.levels = c(0.9,0.95,0.99), col.contour = c("dark
blue","blue","light blue"), ref = exp(ICI_OS$TE.fixed))
legend("topleft", c("p < 0.01", "0.01 < p < 0.05", "0.05 < p < 0.10", "p > 0.10"), fill=c("light
blue","blue","dark blue", 'white'), bg = 'white')
ICIOS.bias <- metabias(ICI_OS2,method.bias="linreg",plotit=T)
ICIOS.bias
abline(h=c(0,-2,2), col = c("red","gray","gray"))

#Meta-regression
Line<-read_xlsx('/Users/kychi/Desktop/ICI and PPI/Pairwise/Sensitivity analysis.xlsx',
       sheet = 'line')
Line_OS<-metagen(TE, seTE, studlab = study, data = line, comb.fixed = F, sm = 'HR',
          method.tau = 'REML')
Line.reg <- metareg (line_OS,~line, method.tau="REML", hakn = TRUE)
bubble (Line.reg, col='pink',bg='orange', col.line='red', xlab = '1st line therapy (%)',
        ylab = 'logHR (PPI vs non-PPI)')
Line.reg
```

Result 1. Studies that were excluded following full-text inspection

Not report the comparative survival outcome of the use of concomitant PPI or H2B in patients receiving ICI or chemotherapy (n=14)

1. *Angrish, M. D., et al. (2021). "Association of Antibiotics and Other Drugs with Clinical Outcomes in Metastatic Melanoma Patients Treated with Immunotherapy." J Skin Cancer 2021: 9120162.*
2. *Buti, S., et al. (2021). "Predictive ability of a drug-based score in patients with advanced non-small-cell lung cancer receiving first-line immunotherapy." Eur J Cancer 150: 224-231.*
3. *Morales-Barrera, R., et al. (2020). "Effect of concurrent proton pump inhibitors (PPI) use in patients (pts) treated with immune checkpoint inhibitors (ICI) for metastatic urothelial carcinoma (mUC)." Journal of Clinical Oncology 38(6_suppl): 500-500.*
4. *Hamy, A. S., et al. (2020). "Comedications influence immune infiltration and pathological response to neoadjuvant chemotherapy in breast cancer." OncoImmunology 9(1).*
5. *Gandhi, S., et al. (2020). "Impact of concomitant medication use and immune-related adverse events on response to immune checkpoint inhibitors." Immunotherapy **12**(2): 141-149.*
6. *Clémence, B., et al. (2019). "Impact of concomitant medications on disease free survival (DFS) and overall survival (OS) in patients from the PETACC8 study." Annals of Oncology **30**: v207-v208.*
7. *Nguyen, Q. P., et al. (2019). "The effect of proton pump inhibitors on the efficacy of nivolumab monotherapy in different types of cancer." Annals of Oncology **30**: vi115.*
8. *Trabolsi, A., et al. (2019). "Proton pump inhibitors and response to immune check-point inhibitors: Single center study." Journal of Clinical Oncology **37**(15_suppl): e14092-e1409.*
9. *Weinstock, C., et al. (2019). "Impact of antibiotic use on clinical outcomes in patients with urothelial cancer receiving a programmed death protein 1 or programmed death ligand 1 (anti-PD-1/L1) antibody." Journal of Clinical Oncology **37**(15_suppl): 4557-4557.*
10. *Kulkarni, A., et al. (2019). "Impact of antibiotics and proton pump inhibitors on clinical outcomes of immune check point blockers in advanced non-small cell lung cancers and metastatic renal cell cancer." Journal of Clinical Oncology **37**(15_suppl): e20520-e20520.*
11. *Homicsko, K., et al. (2018). "Proton pump inhibitors negatively impact survival of PD-1 inhibitor based therapies in metastatic melanoma patients." Annals of Oncology **29**: x40.*
12. *Hamilton, R. J., et al. (2014). "Effect of concomitant medication use on outcomes of treatment and placebo arms of the COU-AA-301 and COU-AA-302 studies of abiraterone acetate (AA) in metastatic castration-resistant prostate cancer (mCRPC)." Journal of Clinical Oncology 32(15_suppl): e16045-e16045.*
13. *Li B, Cao F, Zhu Q, Li B, Gan M, Wang D. Perioperative cimetidine administration improves systematic immune response and tumor infiltrating lymphocytes in patients with colorectal cancer. Hepatogastroenterology. 2013 Mar-Apr;60(122):244-7. doi: 10.5754/hge12573. PMID: 22944376.*
14. *Scheulen ME, Saito K, Hilger RA, Mende B, Zergebel C, Strumberg D. Effect of food and a proton pump inhibitor on the pharmacokinetics of S-1 following oral administration of S-1 in patients with advanced solid tumors. Cancer Chemother Pharmacol. 2012 Mar;69(3):753-61. doi: 10.1007/s00280-011-1761-2.*

Study did not separate the survival outcome of PPI and H2B in patients receving ICI or chemotherapy (n=3)

1. *Hakozaki, T., et al. (2019). "Impact of prior antibiotic use on the efficacy of nivolumab for non-small cell lung cancer." Oncol Lett **17**(3): 2946-2952.*

2. *Rhinehart, H. E., et al. (2019). "Evaluation of the clinical impact of concomitant acid suppression therapy in colorectal cancer patients treated with capecitabine monotherapy." J Oncol Pharm Pract **25**(8): 1839-1845.*

3. *Ali AH, Hale L, Yalamanchili B, Ahmed M, Ahmed M, Zhou R, Wright SE. The Effect of Perioperative Cimetidine Administration on Time to Colorectal Cancer Recurrence. Am J Ther. 2018 Jul/Aug;25(4):e405-e411. doi: 10.1097/MJT.0000000000000547. PMID: 29630589.*

Patients receiving concurrent radiotherapy (n=5)

1. *Tvingsholm SA, Dehlendorff C, Østerlind K, Friis S, Jäättelä M. Proton pump inhibitor use and cancer mortality. Int J Cancer. 2018 Sep 15;143(6):1315-1326. doi: 10.1002/ijc.31529. Epub 2018 May 2. PMID: 29658114*

2. *Kearns MD, Boursi B, Yang YX. Proton pump inhibitors on pancreatic cancer risk and survival. Cancer Epidemiol. 2017 Feb;46:80-84. doi: 10.1016/j.canep.2016.12.006. Epub 2017 Jan 2. PMID: 28056391; PMCID: PMC5303431*

3. *Lalani AA, McKay RR, Lin X, Simantov R, Kaymakcalan MD, Choueiri TK. Proton Pump Inhibitors and Survival Outcomes in Patients With Metastatic Renal Cell Carcinoma. Clin Genitourin Cancer. 2017 Dec;15(6):724-732. doi: 10.1016/j.clgc.2017.05.019. Epub 2017 May 31. PMID: 28645482.*

4. *Zhang, J. L., et al. (2017). "Effects of omeprazole in improving concurrent chemoradiotherapy efficacy in rectal cancer." World J Gastroenterol **23**(14): 2575-2584.*

5. *Papagerakis, S., et al. (2014). "Proton pump inhibitors and histamine 2 blockers are associated with improved overall survival in patients with head and neck squamous carcinoma." Cancer Prev Res (Phila) 7(12): 1258-1269.*

Not baseline use of PPI (n=2)

1. *oberto, M., et al. (2020). "Combination therapy of high-dose rabeprazole plus metronomic capecitabine in advanced gastrointestinal cancer: A randomized phase II trial." Cancers 12(11): 1-12.*

2. *Wang, B. Y., et al. (2015). "Intermittent high dose proton pump inhibitor enhances the antitumor effects of chemotherapy in metastatic breast cancer." J Exp Clin Cancer Res 34(1): 85.*

Results 2. The Risk Of Bias In Non-randomized Studies – of Interventions (ROBINS-I) assessment tool

We used the ROBINS-I template provided by ROBINS-I detailed guidance (2016) to steer us toward the critical appraisal of included non-RCTs in our systematic review with meta-analysis regarding our review question. The details are presented in the following sections.

ROBINS-I tool (Stage I): At protocol stage

Specify the review question

Participants	*Cancer patients receiving immunecheckpoint inhibitors*
Experimental intervention	*With baseline gastric acid suppressants*
Comparator	*Without baseline use of gastric acid suppressants*
Outcomes	*Overall survival and progression-free survival between users and non-users*

List the confounding domains relevant to all or most studies

Age, ECOG-PS, PDL-1 expression, peptic ulcer disease, gastroesophageal reflux, coronary artery disease, first line treatment

List co-interventions that could be different between intervention groups and that could impact on outcomes

nil

ROBINS-I tool (Stage II): For each study

Specify a target randomized trial specific to the study

Design	Individually randomized / Cluster randomized / Matched (e.g. cross-over)
Participants	*Cancer patients receiving immune checkpoint inhibitors or chemotherapy*
Experimental intervention	*With concomitant proton pump inhibitors*
Comparator	*Without concomitant proton pump inhibitors*

Is your aim for this study…?

- ☞ to assess the effect of *assignment to* intervention
- ☞ to assess the effect of *starting and adhering to* intervention

Specify the outcome

Specify which outcome is being assessed for risk of bias (typically from among those earmarked for the Summary of Findings table). Specify whether this is a proposed benefit or harm of intervention.

Overall survival and progression-free survival

Specify the numerical result being assessed

In case of multiple alternative analyses being presented, specify the numeric result (e.g. RR = 1.52 (95% CI 0.83 to 2.77) and/or a reference (e.g. to a table, figure or paragraph) that uniquely defines the result being assessed.

Hazard Ratios

Preliminary consideration of confounders

Complete a row for each important confounding domain (i) listed in the review protocol; and (ii) relevant to the setting of this particular study, or which the study authors identified as potentially important.

"Important" confounding domains are those for which, in the context of this study, adjustment is expected to lead to a clinically important change in the estimated effect of the intervention. "Validity" refers to whether the confounding variable or variables fully measure the domain, while "reliability" refers to the precision of the measurement (more measurement error means less reliability).

**(i) Confounding domains listed in the review protocol**				
Confounding domain	Measured variable(s)	Is there evidence that controlling for this variable was unnecessary?*	Is the confounding domain measured validly and reliably by this variable (or these variables)?	OPTIONAL: Is failure to adjust for this variable (alone) expected to favour the experimental intervention or the comparator?
Baseline confounding	Age	Yes	Yes	Favour comparator
	ECOG-PS	Yes	Yes	Favour comparator
	PDL-1 expression	Yes	No information	No information
	Peptic ulcer disease	Yes	Yes	Favour comparator
	Gastroesophageal reflux	Yes	Yes	Favour comparator
	Coronary artery disease	Yes	Yes	Favour comparator

(ii) Additional confounding domains relevant to the setting of this particular study, or which the study authors identified as important				
Confounding domain	Measured variable(s)	Is there evidence that controlling for this variable was unnecessary?*	Is the confounding domain measured validly and reliably by this variable (or these variables)?	OPTIONAL: Is failure to adjust for this variable (alone) expected to favour the experimental intervention or the comparator?
Baseline confounding	First line treatment	No	Yes	No information

* In the context of a particular study, variables can be demonstrated not to be confounders and so not included in the analysis: (a) if they are not predictive of the outcome; (b) if they are not predictive of intervention; or (c) because adjustment makes no or minimal difference to the estimated effect of the primary parameter. Note that "no statistically significant association" is not the same as "not predictive".

Preliminary consideration of co-interventions

Complete a row for each important co-intervention (i) listed in the review protocol; and (ii) relevant to the setting of this particular study, or which the study authors identified as important.

"Important" co-interventions are those for which, in the context of this study, adjustment is expected to lead to a clinically important change in the estimated effect of the intervention.

(i) Co-interventions listed in the review protocol		
Co-intervention	Is there evidence that controlling for this co-intervention was unnecessary (e.g. because it was not administered)?	Is presence of this co-intervention likely to favour outcomes in the experimental intervention or the comparator
Nil	-	-

(ii) Additional co-interventions relevant to the setting of this particular study, or which the study authors identified as important		
Co-intervention	Is there evidence that controlling for this co-intervention was unnecessary (e.g. because it was not administered)?	Is presence of this co-intervention likely to favour outcomes in the experimental intervention or the comparator
Nil	-	-

• Justification of risk of bias of each study in each domain

Study	Description	Risk of bias
Domain 1: Bias due to confounding		
Alessio 2021	There was a lack of details regarding the baseline demographics between PPI users and PPI non-users. However, the study did conduct multivariate analysis of OS and PFS for significant variables (i.e., baseline steroids, antibiotics or PPI) in univariate analysis.	Moderate
Buti 2021	The study was not only scarce of baseline characteristics between PPI users and PPI non-users but of adjusted analysis for significant variables.	Serious
Gaucher 2021	There was a lack of details regarding the baseline demographics between PPI users and PPI non-users. However, the study did conduct multivariate analysis regarding baseline use of PPI.	Moderate
Jun 2021	There was significant different in the use of antibiotics and steroids between PPI users and non-users and the study did not perform adjusted analysis regarding PPI use.	Serious
Muira 2021	There was a lack of details regarding the baseline demographics between PPI users and PPI non-users. However, the study did conduct multivariate analysis of OS for significant variables (i.e., baseline use of opioids and PPI) in univariate analysis.	Moderate
Rounis 2021	The study was not only scarce of baseline characteristics between PPI users and PPI non-users but of adjusted analysis for significant variables.	Serious
Kostine 2021	The study was not only scarce of baseline characteristics between PPI users and PPI non-users but of adjusted analysis for significant variables.	Serious
Peng 2021	There was significant difference in ECOG performance status between PPI users and non-users. However, the study did conduct multivariate analysis regarding baseline use of PPI.	Moderate
Bañobre 2021	There was a lack of details regarding the baseline demographics between PPI users and PPI non-users. However, the study did conduct multivariate analysis of OS and PFS for significant variables (i.e., baseline use of antibiotics and PPI) in univariate analysis.	Moderate
Alessio 2020	There was significant difference in ECOG performance status between PPI users and non-users. However, the study did conduct multivariate analysis regarding baseline use of PPI.	Moderate
Ruiz 2020	The study was not only scarce of baseline characteristics between PPI users and PPI non-users but of adjusted analysis for significant variables.	Serious
Estevez 2020	There was a lack of details regarding the baseline demographics between PPI users and PPI non-users. However, the study did conduct multivariate analysis regarding baseline use of PPI.	Moderate
Svaton 2020	There was a lack of details regarding the baseline demographics between PPI users and PPI non-users. However, the study did conduct multivariate analysis regarding baseline use of PPI.	Moderate
Santamaria 2020	There was a lack of details regarding the baseline demographics between PPI users and PPI non-users. However, the study did conduct multivariate analysis of OS and PFS (i.e., baseline use of antibiotics, steroids and PPI) in univariate analysis	Moderate
Wong 2019	There was a lack of details regarding the baseline demographics between PPI users and PPI non-users. However, the study did conduct multivariate analysis regarding baseline use of PPI.	Moderate

Wang 2017	The study was not only scarce of baseline characteristics between PPI users and PPI non-users but of adjusted analysis for significant variables.	Serious
Failing 2016	The study was not only scarce of baseline characteristics between PPI users and PPI non-users but of adjusted analysis for significant variables.	Serious
**Domain 2: Bias in selection of participants into the study**		
Failing 2016	The study exclusively selected patients receiving first line of immunotherapy into the survival analysis.	Moderate
**Domain 3: Bias in classification of interventions**		
Buti 2021	The study obtained the use of PPI by reviewing the clinical records; however, PPI use window was not defined.	Moderate
Muira 2021	The study obtained the use of PPI by reviewing the clinical records; however, PPI use window was not defined.	Moderate
Alessio 2021	The study obtained the use of PPI by reviewing the clinical records; however, PPI use window was not defined.	Moderate
Alessio 2020	The study obtained the use of PPI by reviewing the clinical records; however, PPI use window was not defined.	Moderate
Failing 2016	The study obtained the use of PPI by reviewing the clinical records; however, PPI use window was not defined.	Moderate
Sun 2016	The study obtained the use of PPI by reviewing the clinical records; however, PPI use window was not defined.	Moderate

**Domain 4: Bias due to deviations from intended interventions**		
**If your aim for this study is to assess the effect of assignment to intervention, answer questions 4.1 and 4.2**		
4.1. Were there deviations from the intended intervention beyond what would be expected in usual practice?		N
**Risk of bias judgement**		Low
**Domain 5: Bias due to missing data**		
5.1 Were outcome data available for all, or nearly all, participants?		Y
5.2 Were participants excluded due to missing data on intervention status?		N
5.3 Were participants excluded due to missing data on other variables needed for the analysis?		N
**Risk of bias judgement**		Low
**Domain 6: Bias in measurement of outcomes**		
6.1 Could the outcome measure have been influenced by knowledge of the intervention received?		N
6.2 Were outcome assessors aware of the intervention received by study participants?		Y
6.3 Were the methods of outcome assessment comparable across intervention groups?		Y
6.4 Were any systematic errors in measurement of the outcome related to intervention received?		N
**Risk of bias judgement**		Low
**Domain 7: Bias in selection of the reported result**		
Is the reported effect estimate likely to be selected, on the basis of the results, from...		
7.1. ... multiple outcome *measurements* within the outcome domain?		N
7.2 ... multiple *analyses* of the intervention-outcome relationship?		N
7.3 ... different *subgroups*?		N
**Risk of bias judgement**		Low

The risk of bias was evaluated as low through domain 4 to domain 7 across included

•Figure S1. Visual summary of risk of bias by the use of ROBINS-I evaluation tool.

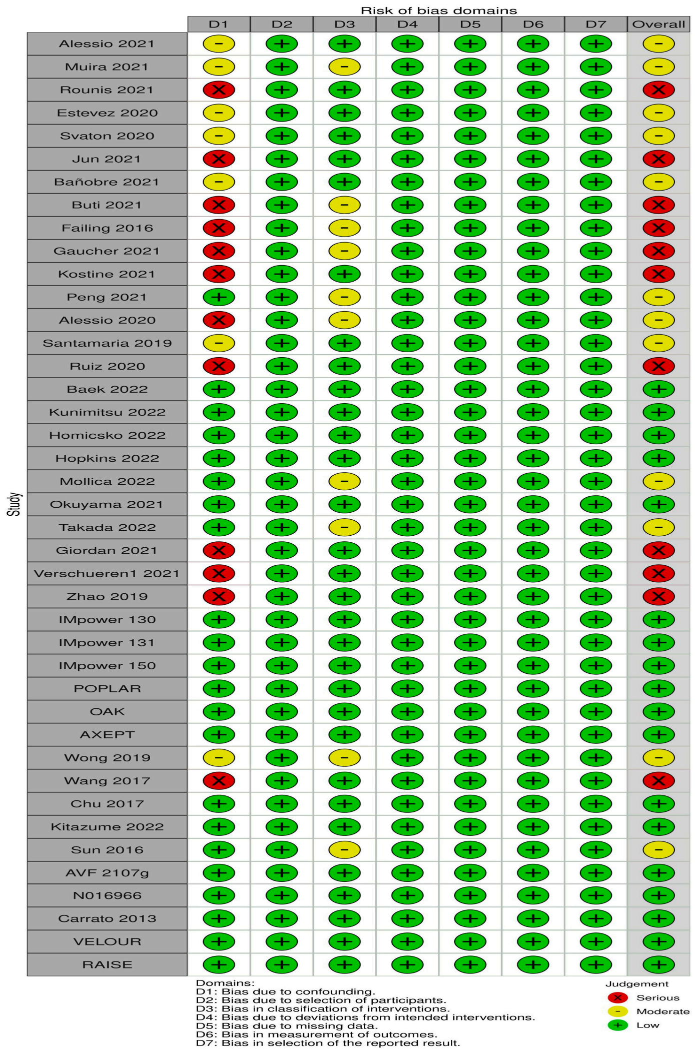

Result 3. Details of NMA of OS and PFS

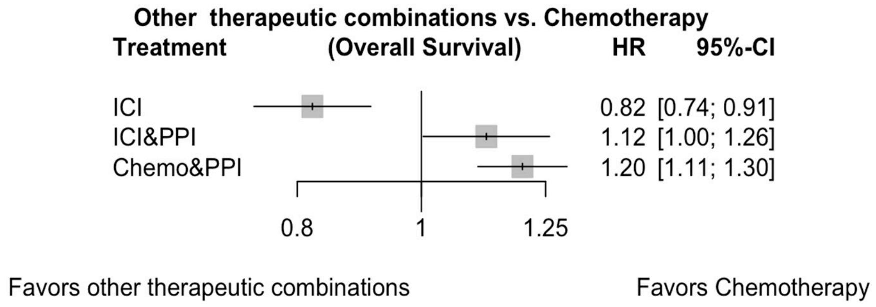

Figure S2. Forest plot of network estimates for hazard ratio (HR) of overall survival with chemotherapy as reference.

Details of network meta-analysis:

```
Number of studies: k = 43
Number of treatments: n = 4
Number of pairwise comparisons: m = 43
Number of designs: d = 1

Random effects model

Treatment estimate (sm = 'HR', comparison: other treatments vs 'Chemotherapy'):
              HR          95%-CI     z  p-value
Chemo&PPI   1.1990 [1.1067; 1.2990]  4.44 < 0.0001
Chemotherapy      .             .     .      .
ICI         0.8220 [0.7401; 0.9130] -3.66   0.0003
ICI&PPI     1.1237 [1.0031; 1.2588]  2.01   0.0440

Quantifying heterogeneity:
tau^2 = 0.0159; tau = 0.1260; I^2 = 49% [26.7%; 64.5%]

Test of heterogeneity:
     Q d.f. p-value
 78.46   40  0.0003
```

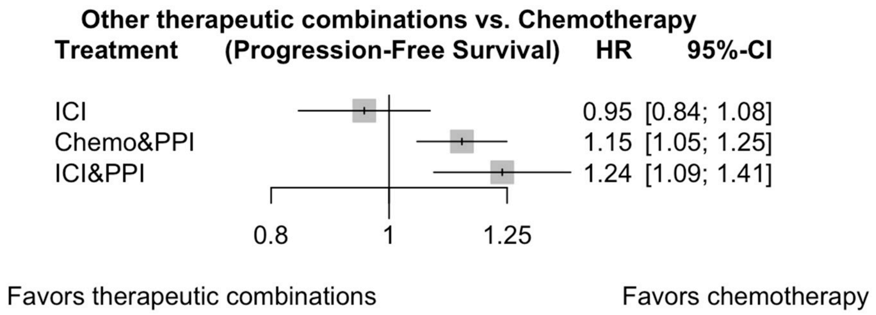

Figure S3. Forest plot of network estimates for hazard ratio (HR) of progression-free survival with chemotherapy as reference.

Details of network meta-analysis:

```
Number of studies: k = 37
Number of treatments: n = 4
Number of pairwise comparisons: m = 37
Number of designs: d = 1

Random effects model

Treatment estimate (sm = 'HR', comparison: other treatments vs 'Chemotherapy'):
              HR          95%-CI     z p-value
Chemo&PPI   1.1477 [1.0542; 1.2494]  3.18  0.0015
Chemotherapy      .             .     .     .
ICI         0.9541 [0.8425; 1.0806] -0.74  0.4596
ICI&PPI     1.2386 [1.0883; 1.4096]  3.24  0.0012

Quantifying heterogeneity:
tau^2 = 0.0201; tau = 0.1419; I^2 = 60.3% [42.6%; 72.6%]

Test of heterogeneity:
     Q d.f.  p-value
 85.65   34 < 0.0001
```

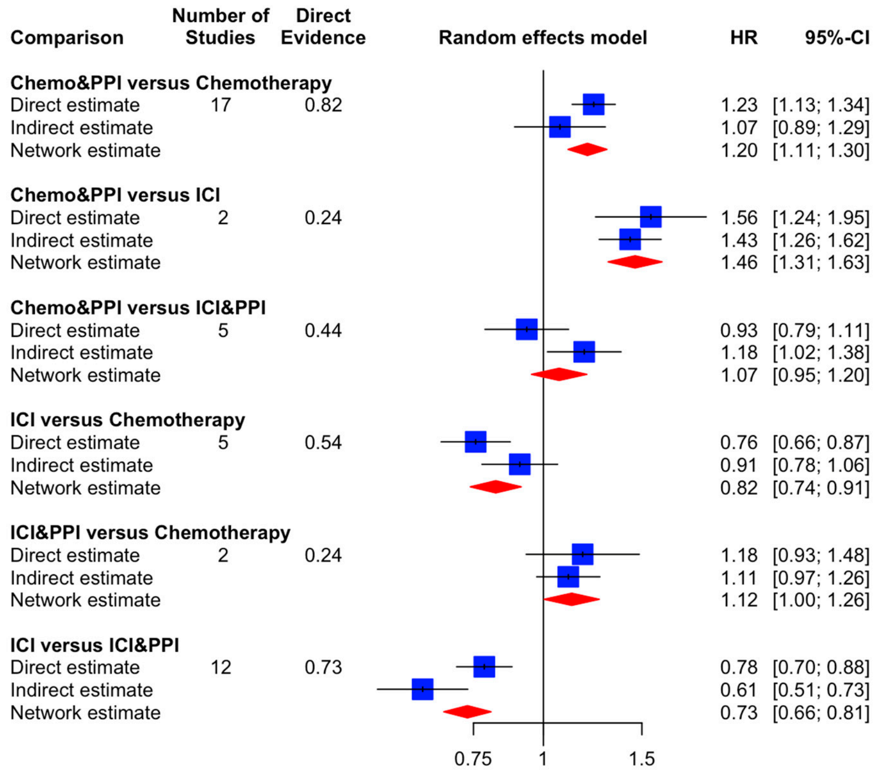

Figure S4. Forest plot of netsplitting direct and indirect network estimates for hazard ratio (HR) of overall survival. No significant inconsistency was demonstrated. ICI, immune checkpoint inhibitors; PPI, proton pump inhibitors; chemo, chemotherapy

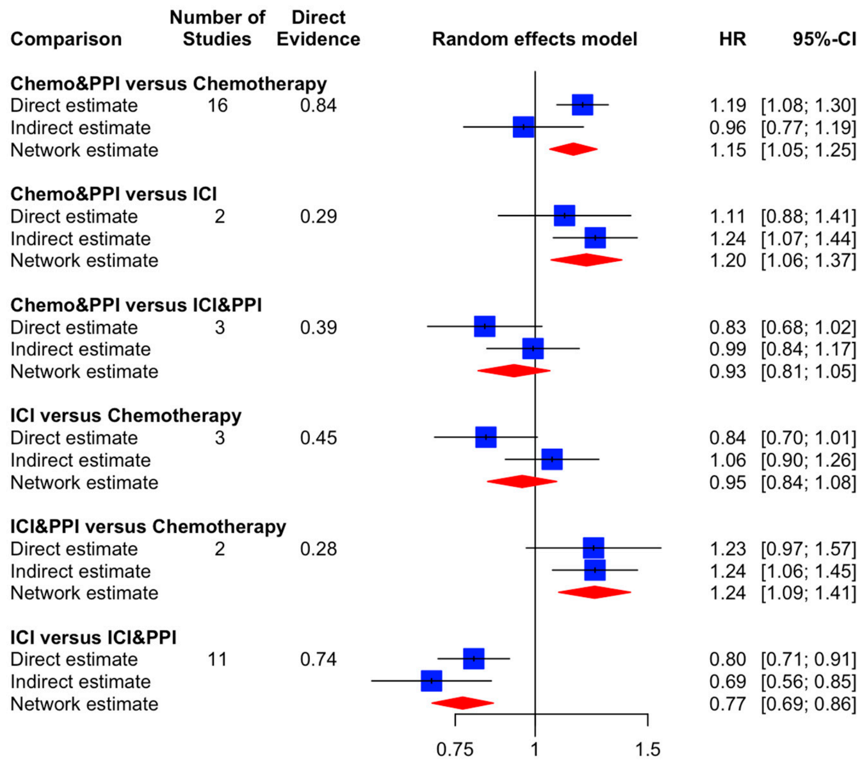

Figure S5. Forest plot of netsplitting direct and indirect network estimates for hazard ratio (HR) of progression-free survival. No significant inconsistency was demonstrated. ICI, immune checkpoint inhibitors; PPI, proton pump inhibitors; chemo, chemotherapy

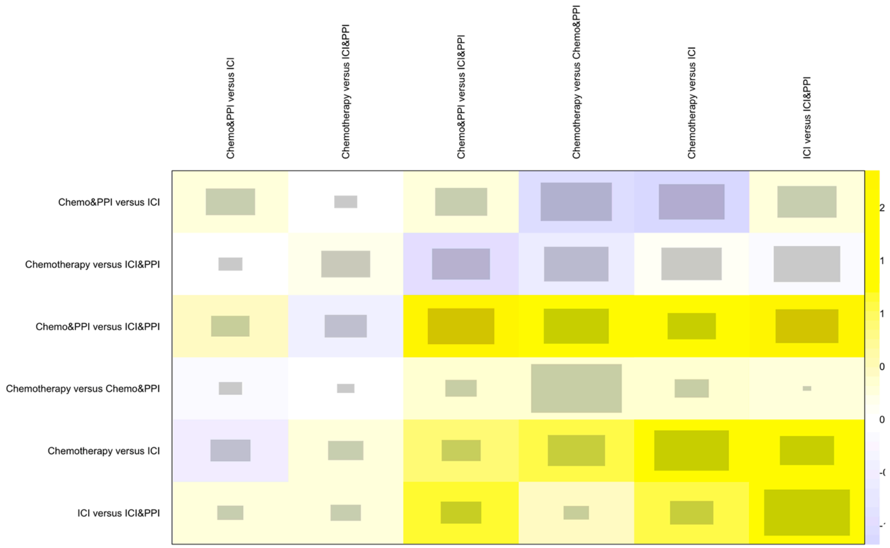

Figure S6. Netheat plot of network estimates for hazard ratio (HR) of overall survival. The gray squares indicate the direct estimates. The colors are associated with the change in inconsistency between direct and indirect comparison. Intense color (eg, red) indicates stronger inconsistency. Through simple visualization, inconsistency can be identified throughout the comparisons. ICI, immune checkpoint inhibitors; PPI, proton pump inhibitors; chemo, chemotherapy

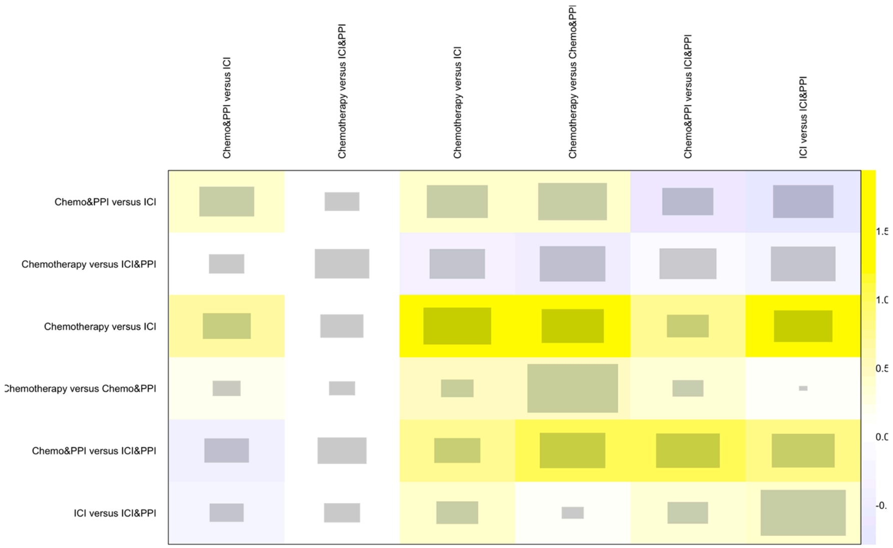

Figure S7. Netheat plot of network estimates for hazard ratio (HR) of progression-free survival. The gray squares indicate the direct estimates. The colors are associated with the change in inconsistency between direct and indirect comparison. Intense color (eg, red) indicates stronger inconsistency. Through simple visualization, inconsistency can be identified throughout the comparisons. ICI, immune checkpoint inhibitors; PPI, proton pump inhibitors; chemo, chemotherapy

Result 4. Network meta-analysis after excluding melanoma trials

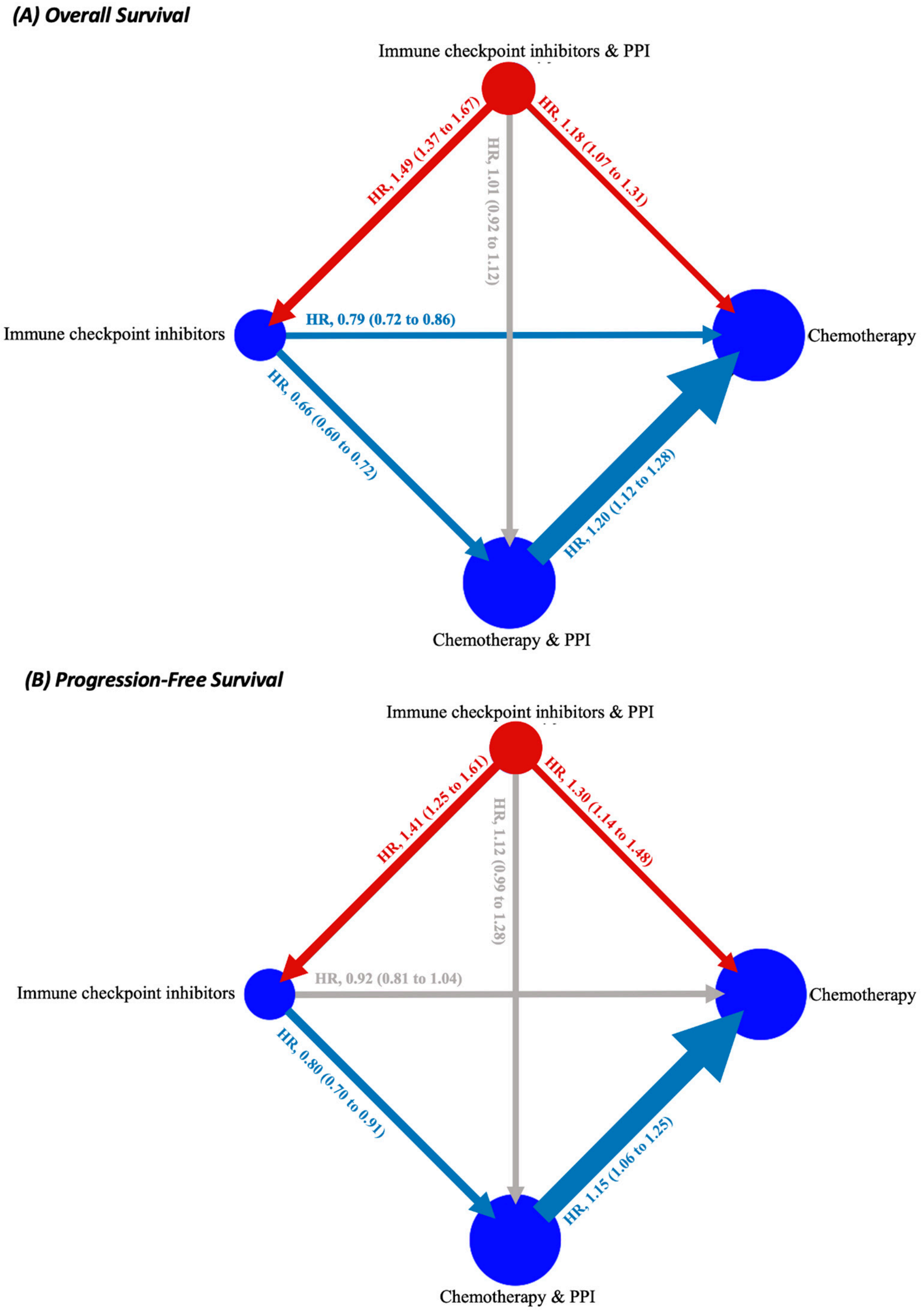

Figure S8. Network plot for comparative association between PPI, ICI, and chemotherapy in terms of (A) overall survival, and (B) progression-free survival.
The thickness of the connecting line corresponds to the number of trials between comparators. We specifically highlight the arm ICI with baseline PPI as red node and the red barrow as its significant comparison with ICI and chemotherapy without baseline PPI to reiterate our main findings. Blue arrows also indicate significant survival association between two nodes. Conversely, gray arrows suggest little association between two arms.
PPI, proton pump inhibitors; ICI, immune checkpoint inhibitors; HR, hazard ratio

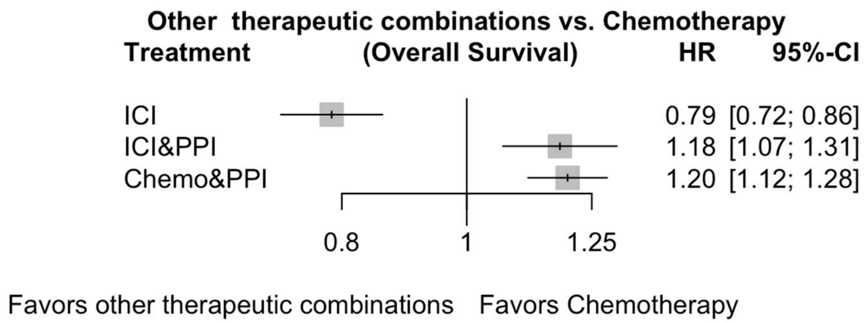

Figure S9. Forest plot of network estimates for hazard ratio (HR) of overall survival with chemotherapy as reference.

Details of network meta-analysis:

Number of studies: k = 36
Number of treatments: n = 4
Number of pairwise comparisons: m = 36
Number of designs: d = 1

Random effects model

Treatment estimate (sm = 'HR', comparison: other treatments vs 'Chemotherapy'):

	HR	95%-CI	z	p-value
Chemo&PPI	1.1972	[1.1155; 1.2850]	4.99	< 0.0001
Chemotherapy	.	.	.	.
ICI	0.7856	[0.7173; 0.8604]	-5.20	< 0.0001
ICI&PPI	1.1809	[1.0665; 1.3075]	3.20	0.0014

Quantifying heterogeneity:
tau^2 = 0.0082; tau = 0.0908; I^2 = 36% [2.8%; 57.9%]

Test of heterogeneity:

Q	d.f.	p-value
51.59	33	0.0207

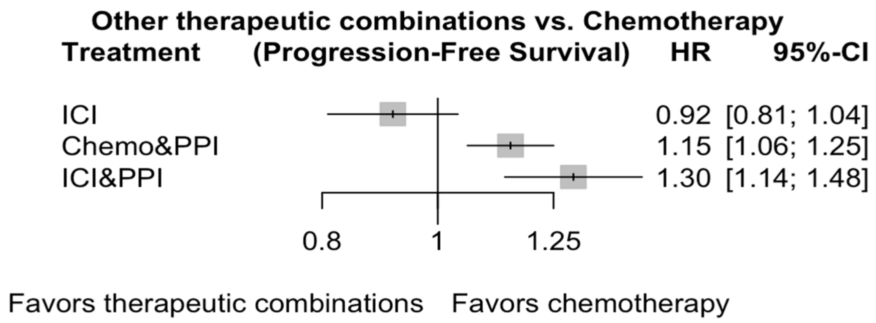

Figure S10. Forest plot of network estimates for hazard ratio (HR) of progression-free survival with chemotherapy as reference.

Details of network meta-analysis:

Number of studies: k = 30
Number of treatments: n = 4
Number of pairwise comparisons: m = 30
Number of designs: d = 1

Random effects model

Treatment estimate (sm = 'HR', comparison: other treatments vs 'Chemotherapy'):

	HR	95%-CI	z	p-value
Chemo&PPI	1.1506	[1.0588; 1.2502]	3.31	0.0009
Chemotherapy	.	.	.	.
ICI	0.9170	[0.8089; 1.0395]	-1.35	0.1757
ICI&PPI	1.2989	[1.1380; 1.4826]	3.87	0.0001

Quantifying heterogeneity:
tau^2 = 0.0187; tau = 0.1366; I^2 = 62.5% [43.6%; 75.0%]

Test of heterogeneity:

Q	d.f.	p-value
71.91	27	< 0.0001

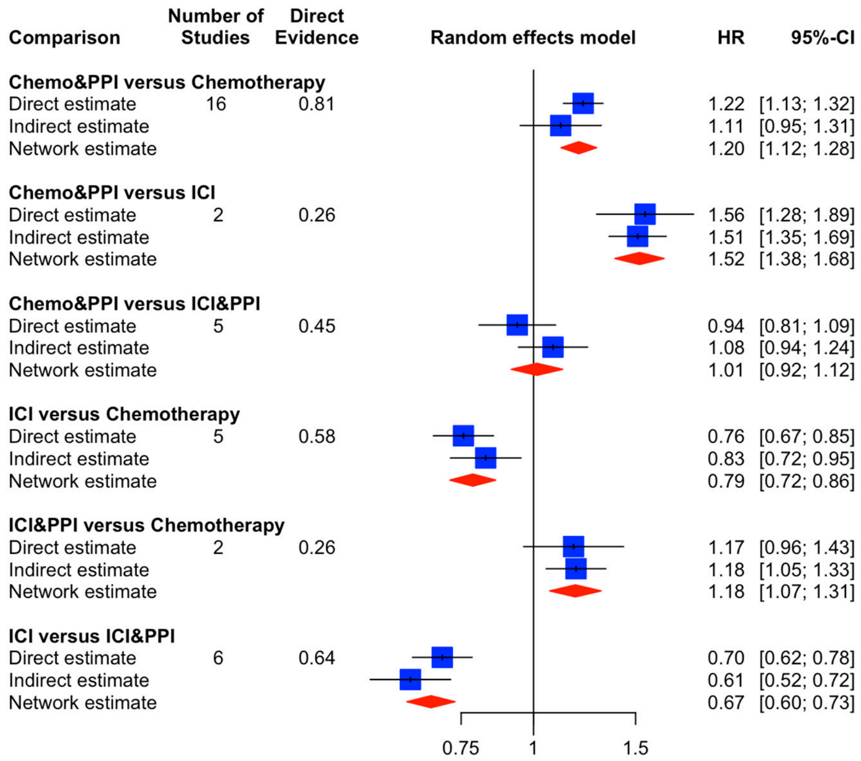

Figure S11. Forest plot of netsplitting direct and indirect network estimates for hazard ratio (HR) of overall survival. No significant inconsistency was demonstrated. ICI, immune checkpoint inhibitors; PPI, proton pump inhibitors; chemo, chemotherapy

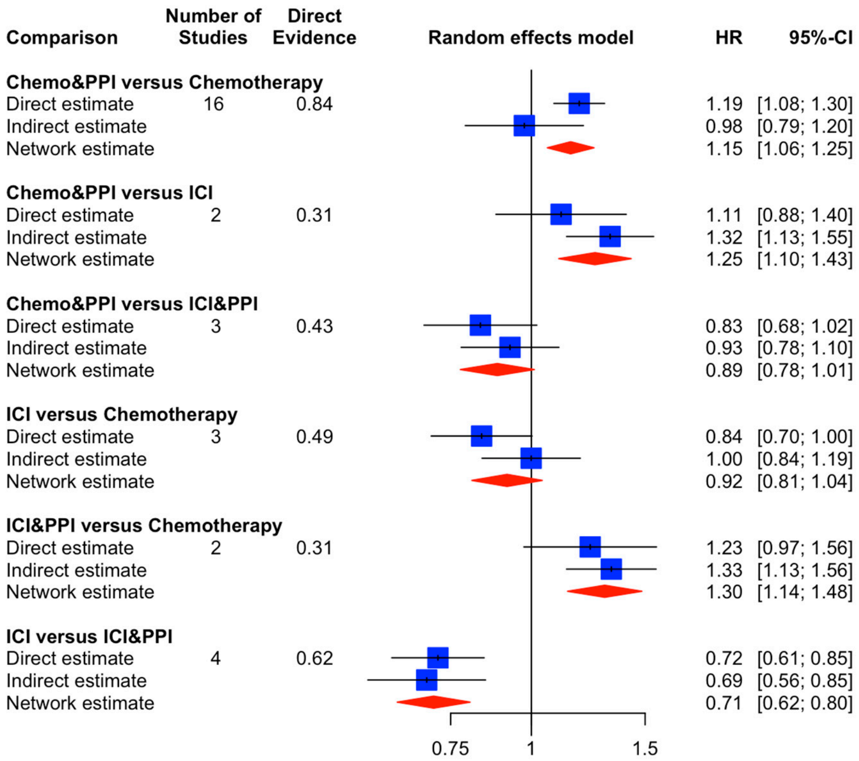

Figure S12. Forest plot of netsplitting direct and indirect network estimates for hazard ratio (HR) of progression-free survival. No significant inconsistency was demonstrated. ICI, immune checkpoint inhibitors; PPI, proton pump inhibitors; chemo, chemotherapy

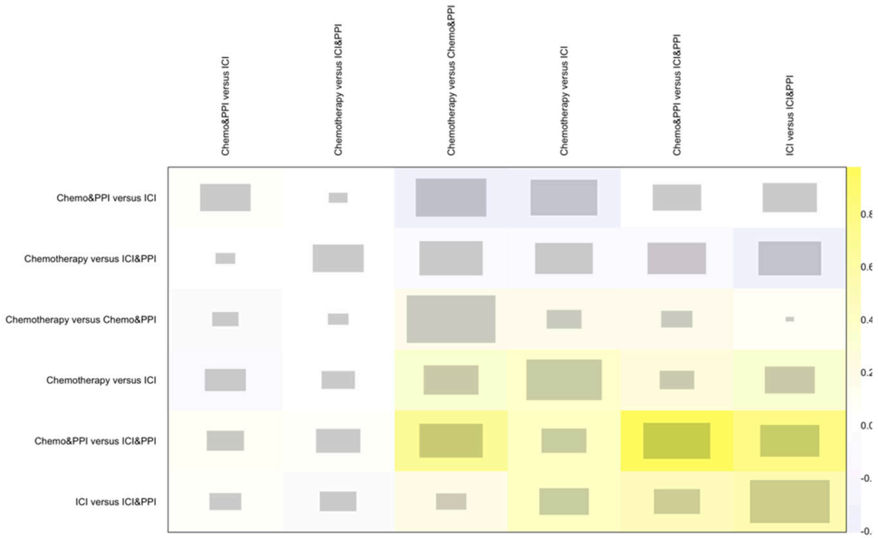

Figure S13. Netheat plot of network estimates for hazard ratio (HR) of overall survival. The gray squares indicate the direct estimates. The colors are associated with the change in inconsistency between direct and indirect comparison. Intense color (eg, red) indicates stronger inconsistency.
No significant inconsistency was demonstrated. ICI, immune checkpoint inhibitors; PPI, proton pump inhibitors; chemo, chemotherapy

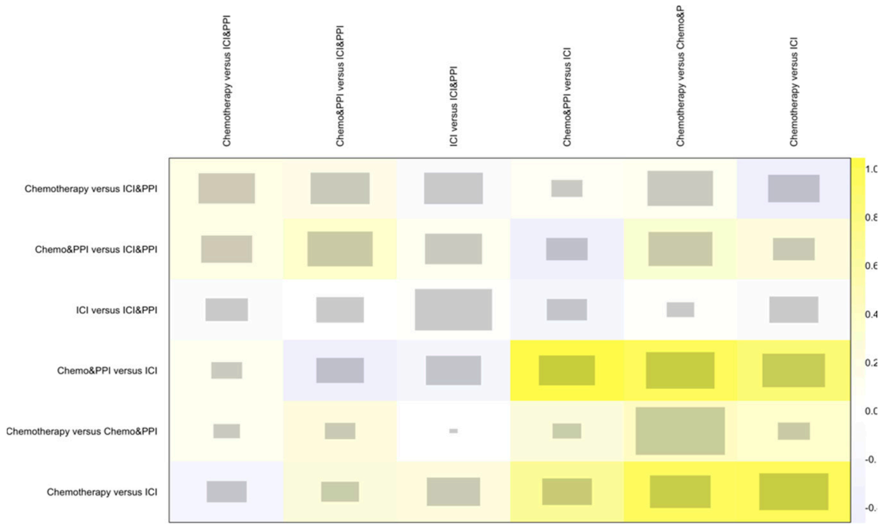

Figure S14. Netheat plot of network estimates for hazard ratio (HR) of progression-free survival. The gray squares indicate the direct estimates. The colors are associated with the change in inconsistency between direct and indirect comparison. Intense color (eg, red) indicates stronger inconsistency.
No significant inconsistency was demonstrated. ICI, immune checkpoint inhibitors; PPI, proton pump inhibitors; chemo, chemotherapy

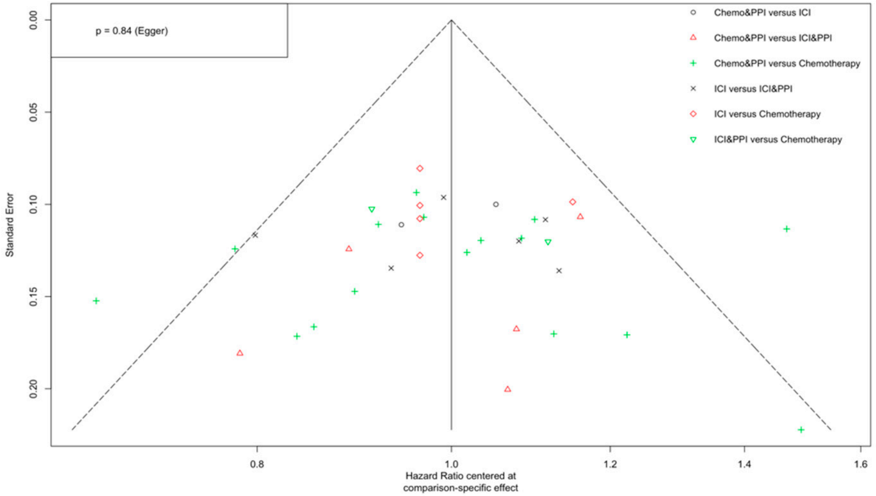

Figure S15. Comparison-adjusted funnel plot. It is symmetrical through inspection and is supported by Egger's test (p=0.84), suggesting the absence of small study effects for overall survival. ICI, immune checkpoint inhibitors; PPI, proton pump inhibitors; chemo, chemotherapy

Linear regression test of funnel plot asymmetry

Test result: t = -0.21, df = 34, p-value = 0.8375

Sample estimates:

bias	se.bias	intercept	se.intercept
-0.1935	0.9362	0.0238	0.1123

Details:
- multiplicative residual heterogeneity variance (tau^2 = 1.4225)
- predictor: standard error
- weight: inverse variance
- reference: Egger et al. (1997), BMJ

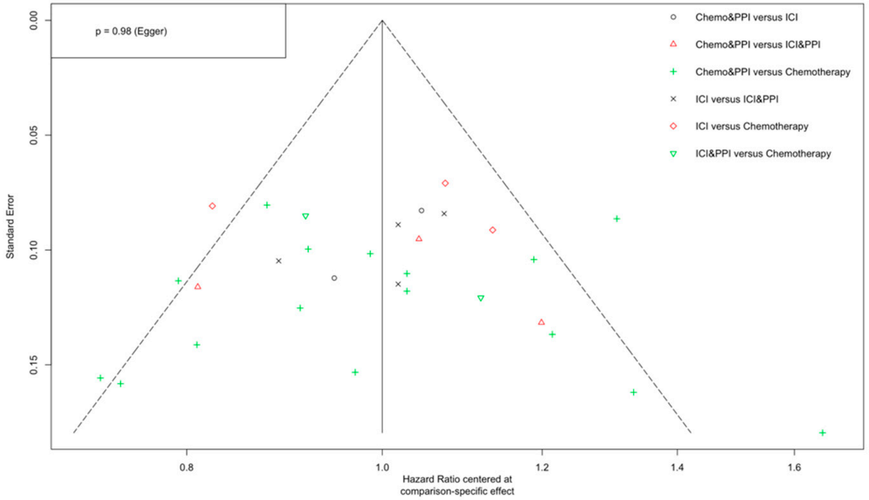

Figure S16. Comparison-adjusted funnel plot. It is symmetrical through inspection and is supported by Egger's test (p=0.98), suggesting the absence of small study effects for progression-free survival. ICI, immune checkpoint inhibitors; PPI, proton pump inhibitors; chemo, chemotherapy

Linear regression test of funnel plot asymmetry

Test result: t = 0.02, df = 28, p-value = 0.9834

Sample estimates:

bias	se.bias	intercept	se.intercept
0.0251	1.1993	-0.0020	0.1253

Details:
- multiplicative residual heterogeneity variance (tau^2 = 2.2237)
- predictor: standard error
- weight: inverse variance
- reference: Egger et al. (1997), BMJ

Result 5. Sensitivity analyses of NMA (excluding studies of 1st line therapy)

(A) Overall Survival

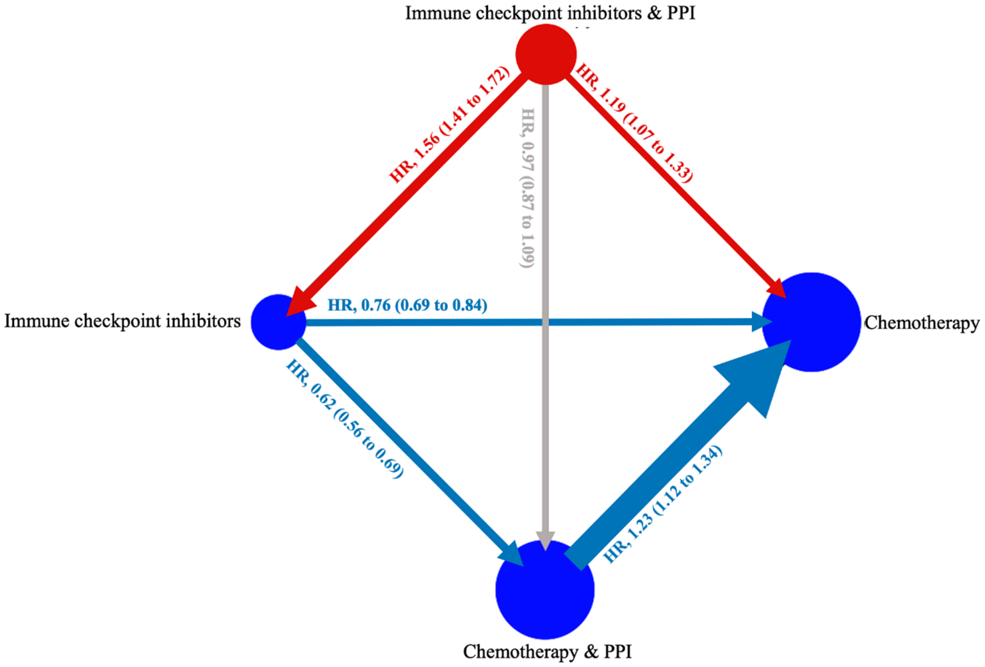

(B) Progression-Free Survival

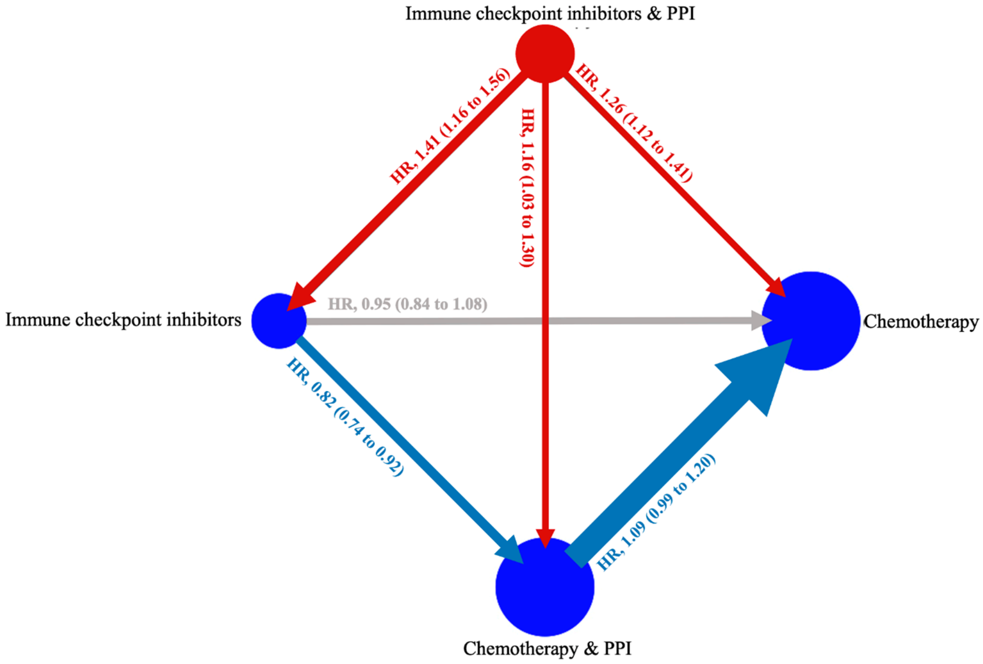

Figure S17. Network plot for comparative association between PPI, ICI, and chemotherapy after excluding first-line therapy in terms of (A) overall survival, and (B) progression-free survival.
The thickness of the connecting line corresponds to the number of trials between comparators. We specifically highlight the arm ICI with baseline PPI as red node and the red barrow as its significant comparison with ICI and chemotherapy without baseline PPI to reiterate our main findings. Blue arrows also indicate significant survival association between two nodes. Conversely, gray arrows suggest little association between two arms.
PPI, proton pump inhibitors; ICI, immune checkpoint inhibitors; HR, hazard ratio

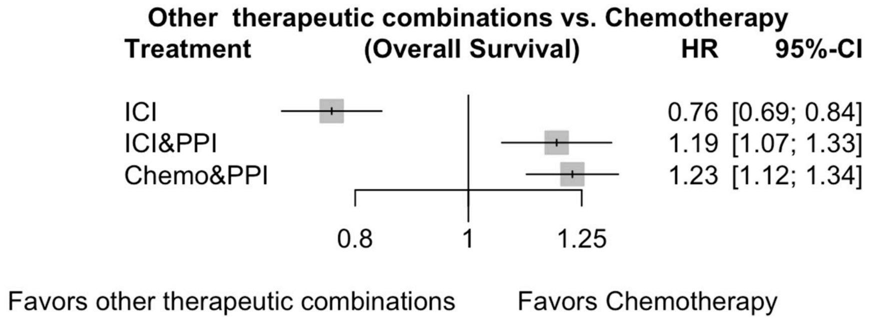

Figure S18. Forest plot of network estimates for hazard ratio (HR) of overall survival after excluding studies of lst line therapy with chemotherapy as reference.

Details of network meta-analysis:

Number of studies: k = 20
Number of treatments: n = 4
Number of pairwise comparisons: m = 20
Number of designs: d = 1

Random effects model

Treatment estimate (sm = 'HR', comparison: other treatments vs 'Chemotherapy'):

	HR [95%-CI]	z	p-value
Chemo&PPI	1.2274 [1.1210; 1.3438]	4.43	< 0.0001
Chemotherapy	.	.	.
ICI	0.7638 [0.6921; 0.8430]	-5.35	< 0.0001
ICI&PPI	1.1899 [1.0679; 1.3258]	3.15	0.0016

Quantifying heterogeneity:
tau^2 = 0.0048; tau = 0.0693; I^2 = 27.7% [0.0%; 59.2%]

Test of heterogeneity:

Q	d.f.	p-value
23.51	17	0.1334

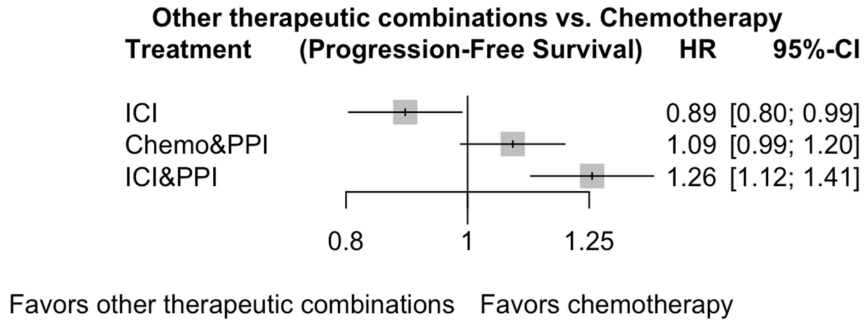

Figure S19. Forest plot of network estimates for hazard ratio (HR) of progression-free survival after excluding studies of lst line therapy with chemotherapy alone as reference.

Details of network meta-analysis:

Number of studies: k = 20
Number of treatments: n = 4
Number of pairwise comparisons: m = 20
Number of designs: d = 1

Random effects model

Treatment estimate (sm = 'HR', comparison: other treatments vs 'Chemotherapy'):

	HR [95%-CI]	z	p-value
Chemo&PPI	1.0865 [0.9875; 1.1954]	1.70	0.0888
Chemotherapy	.	.	.
ICI	0.8919 [0.8038; 0.9896]	-2.16	0.0310
ICI&PPI	1.2568 [1.1228; 1.4067]	3.97	< 0.0001

Quantifying heterogeneity:
tau^2 = 0.0090; tau = 0.0949; I^2 = 48.3% [10.7%; 70.1%]

Test of heterogeneity:

Q	d.f.	p-value
32.89	17	0.0117

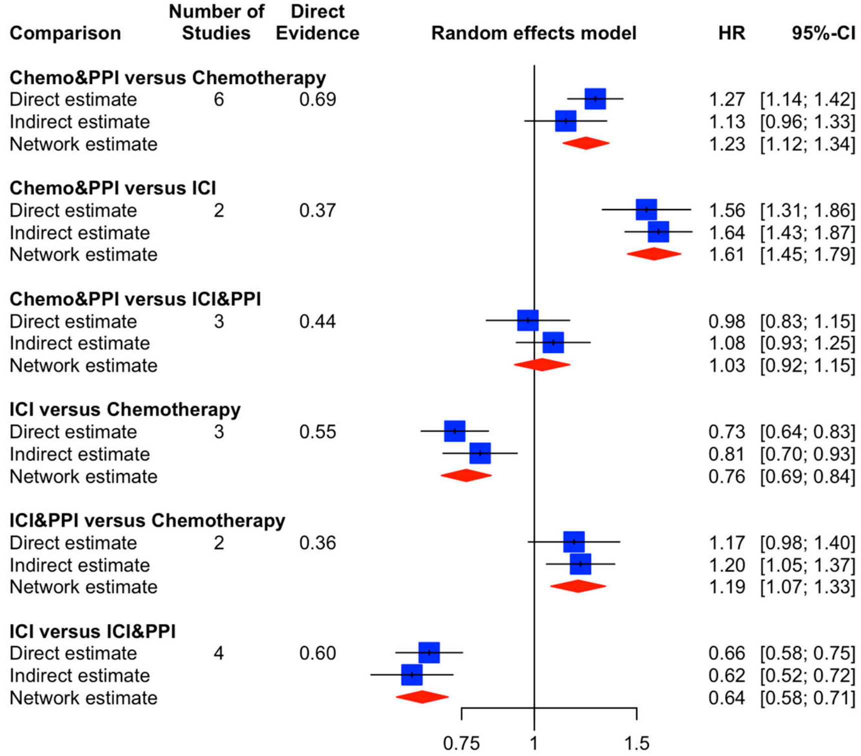

Figure S20. Forest plot of netsplitting direct and indirect network estimates for hazard ratio (HR) of overall survival. No significant inconsistency was demonstrated. ICI, immune checkpoint inhibitors; PPI, proton pump inhibitors; chemo, chemotherapy

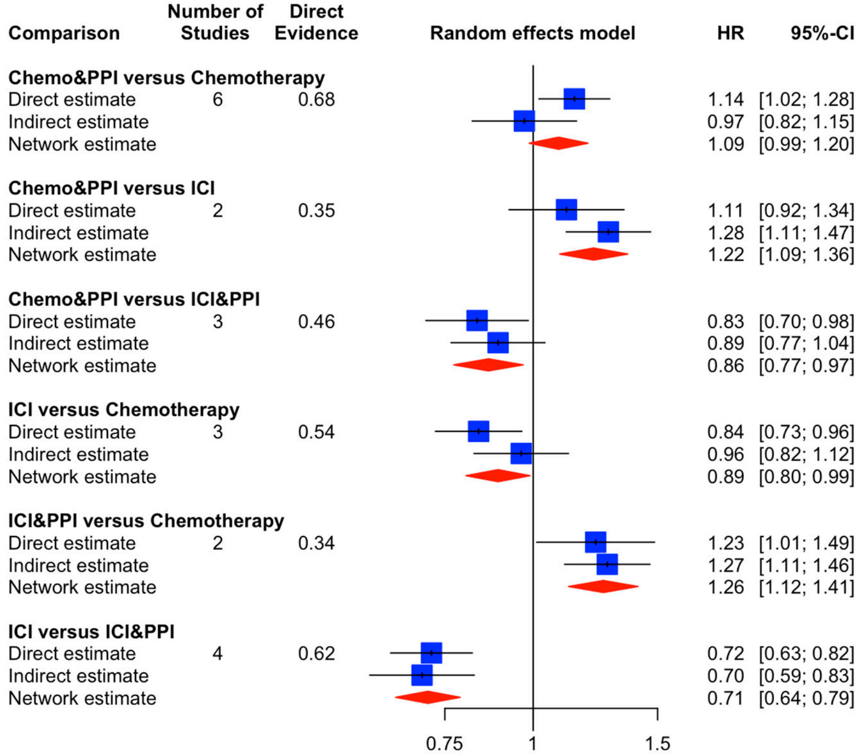

Figure S21. Forest plot of netsplitting direct and indirect network estimates for hazard ratio (HR) of overall survival. No significant inconsistency was demonstrated. ICI, immune checkpoint inhibitors; PPI, proton pump inhibitors; chemo, chemotherapy

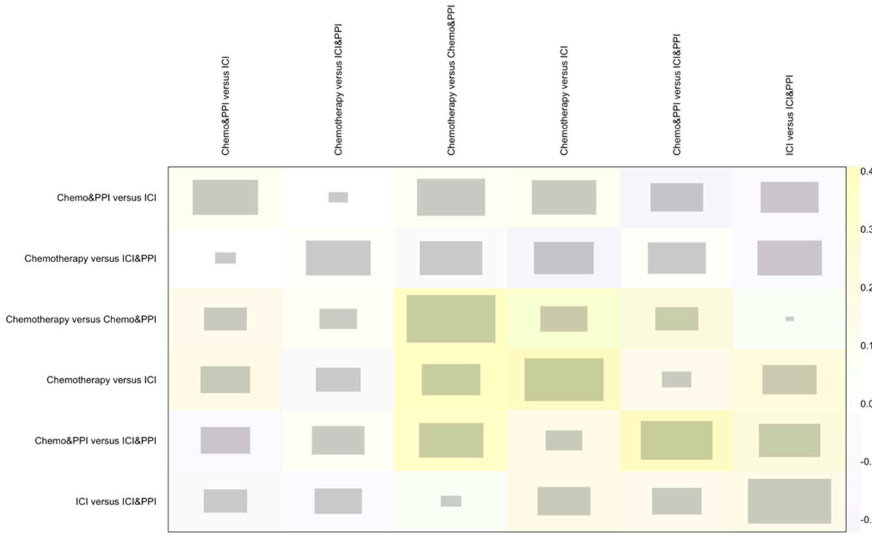

Figure S22. Netheat plot of network estimates for hazard ratio (HR) of overall survival. The gray squares indicate the direct estimates. The colors are associated with the change in inconsistency between direct and indirect comparison. Intense color (eg., red) indicates stronger inconsistency. No significant inconsistency was demonstrated. ICI, immune checkpoint inhibitors; PPI, proton pump inhibitors; chemo, chemotherapy

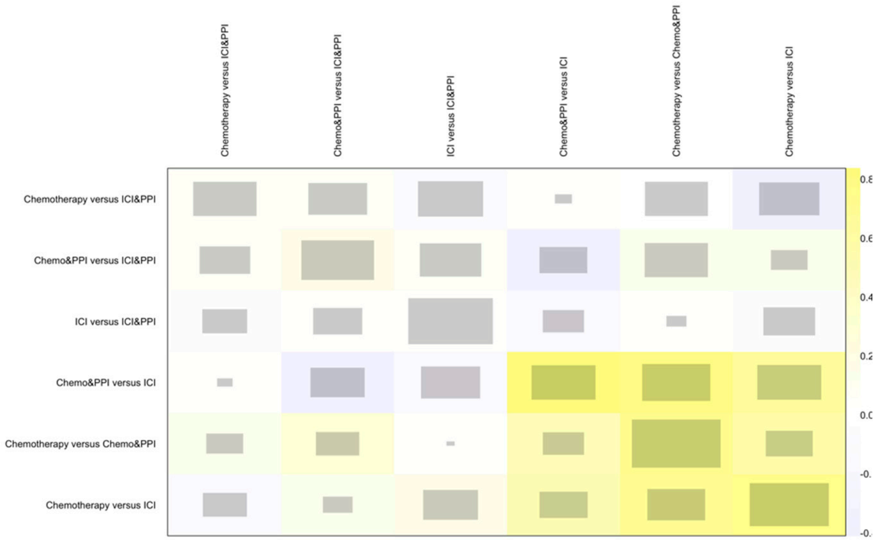

Figure S23. Netheat plot of network estimates for hazard ratio (HR) of progression-free survival. The gray squares indicate the direct estimates. The colors are associated with the change in inconsistency between direct and indirect comparison. Intense color (eg., red) indicates stronger inconsistency. No significant inconsistency was demonstrated. ICI, immune checkpoint inhibitors; PPI, proton pump inhibitors; chemo, chemotherapy

Result 6. Sensitivity analyses of NMA (excluding studies of unknown PDL-1 status)

(A) Overall Survival

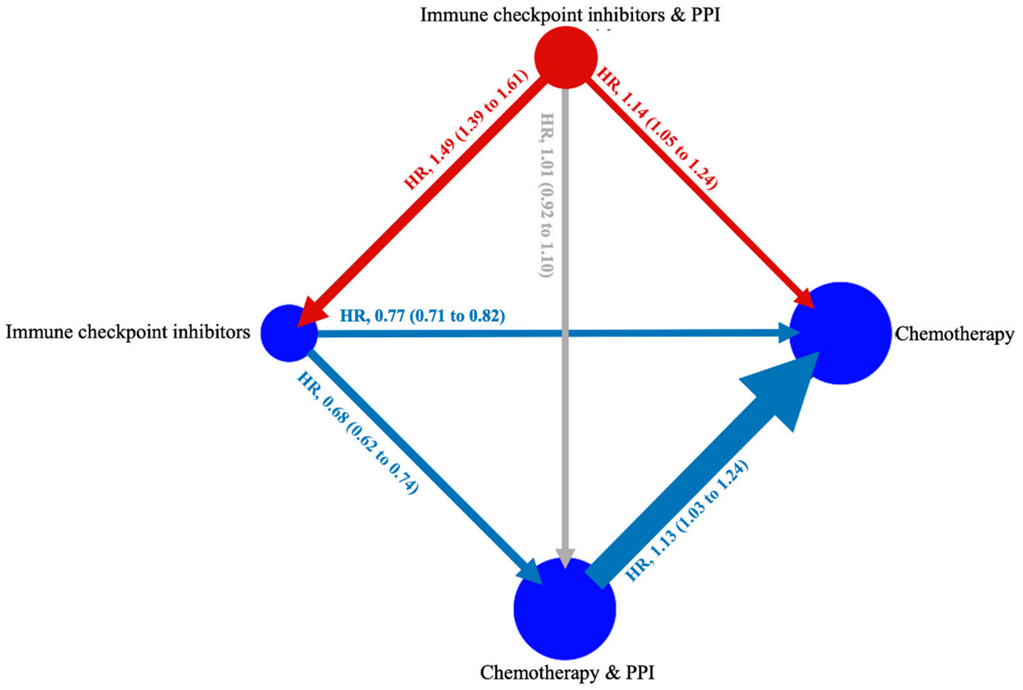

(B) Progression-Free Survival

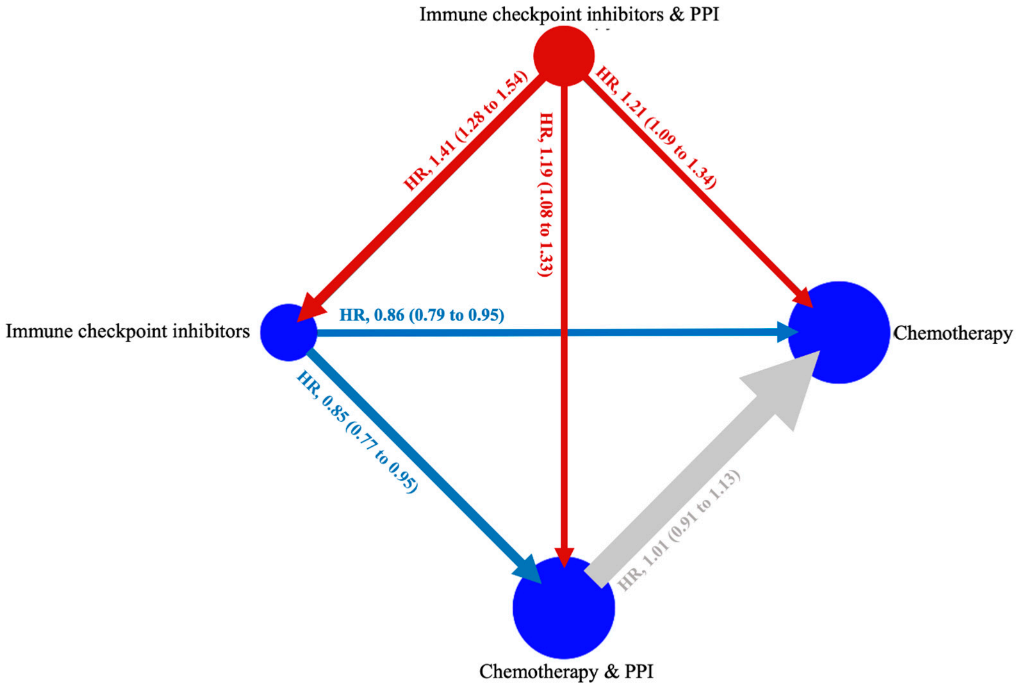

Figure S24. Network plot for comparative association between PPI, ICI, and chemotherapy after excluding unknown PDL-1 status in terms of (A) overall survival, and (B) progression-free survival. The thickness of the connecting line corresponds to the number of trials between comparators. We specifically highlight the arm ICI with baseline PPI as red node and the red barrow as its significant comparison with ICI and chemotherapy without baseline PPI to reiterate our main findings. Blue arrows also indicate significant survival association between two nodes. Conversely, gray arrows suggest little association between two arms.
PPI, proton pump inhibitors; ICI, immune checkpoint inhibitors; HR, hazard ratio

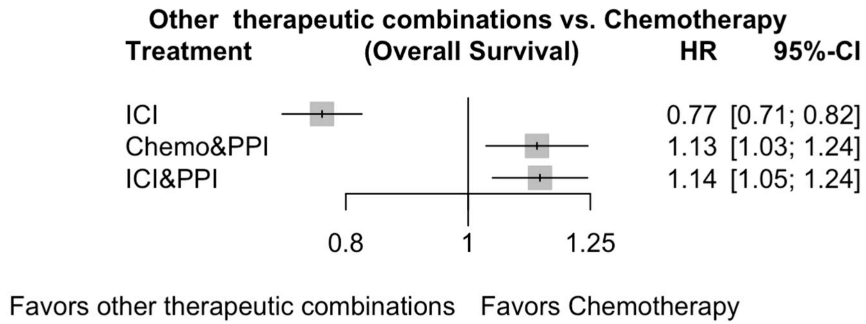

Figure S25. Forest plot of network estimates for hazard ratio (HR) of overall survival after excluding studies of unknown PDL-1 status with chemotherapy as reference.

Details of network meta-analysis:

Number of studies: k = 22
Number of treatments: n = 4
Number of pairwise comparisons: m = 22
Number of designs: d = 1

Random effects model

Treatment estimate (sm = 'HR', comparison: other treatments vs 'Chemotherapy'):

	HR [95%-CI]	z	p-value
Chemo&PPI	1.1339 [1.0333; 1.2444]	2.65	0.0080
Chemotherapy	.	.	.
ICI	0.7658 [0.7121; 0.8237]	-7.18	< 0.0001
ICI&PPI	1.1402 [1.0455; 1.2436]	2.96	0.0030

Quantifying heterogeneity:
tau^2 = 0; tau = 0; I^2 = 0% [0.0%; 48.0%]

Test of heterogeneity:

Q	d.f.	p-value
17.77	19	0.5376

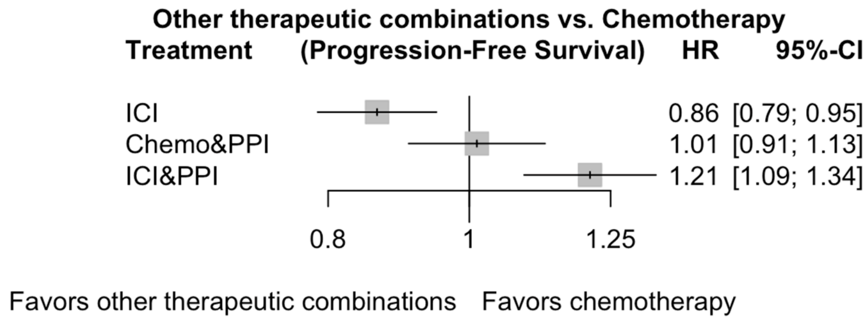

Figure S26. Forest plot of network estimates for hazard ratio (HR) of progression-free survival after excluding studies of unknown PDL-1 status with chemotherapy alone as reference.

Details of network meta-analysis:

Number of studies: k = 16
Number of treatments: n = 4
Number of pairwise comparisons: m = 16
Number of designs: d = 1

Random effects model

Treatment estimate (sm = 'HR', comparison: other treatments vs 'Chemotherapy'):

	HR [95%-CI]	z	p-value
Chemo&PPI	1.0119 [0.9085; 1.1272]	0.22	0.8294
Chemotherapy	.	.	.
ICI	0.8643 [0.7869; 0.9494]	-3.04	0.0023
ICI&PPI	1.2100 [1.0903; 1.3429]	3.59	0.0003

Quantifying heterogeneity:
tau^2 = 0.0048; tau = 0.0692; I^2 = 35% [0.0%; 65.6%]

Test of heterogeneity:

Q	d.f.	p-value
20	13	0.0953

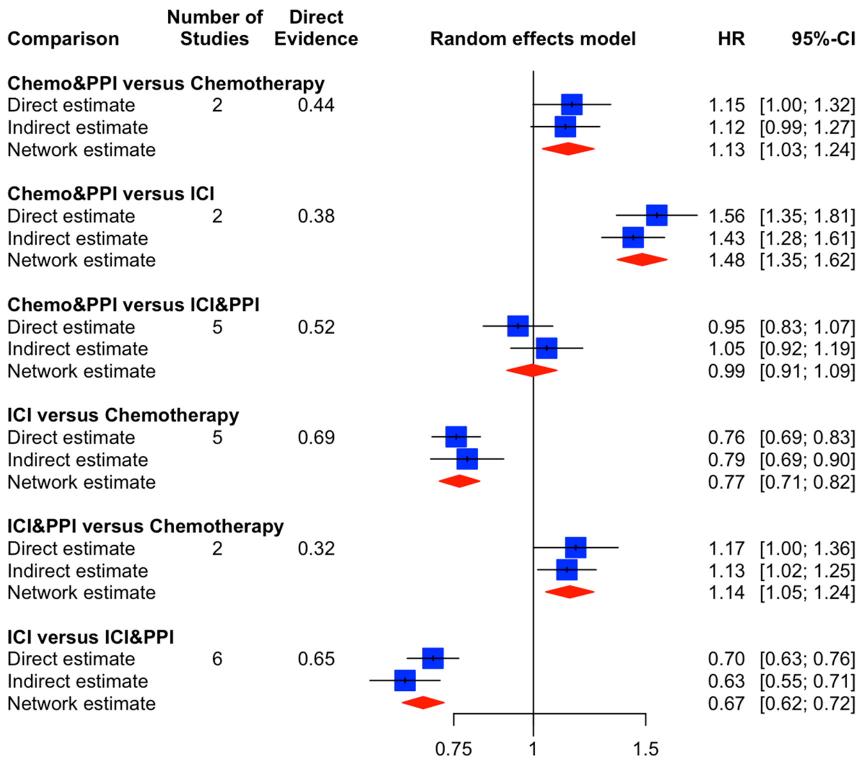

Figure S27. Forest plot of netsplitting direct and indirect network estimates for hazard ratio (HR) of overall survival after excluding studies of unknown PDL-1 status. No significant inconsistency was demonstrated. ICI, immune checkpoint inhibitors; PPI, proton pump inhibitors; chemo, chemotherapy

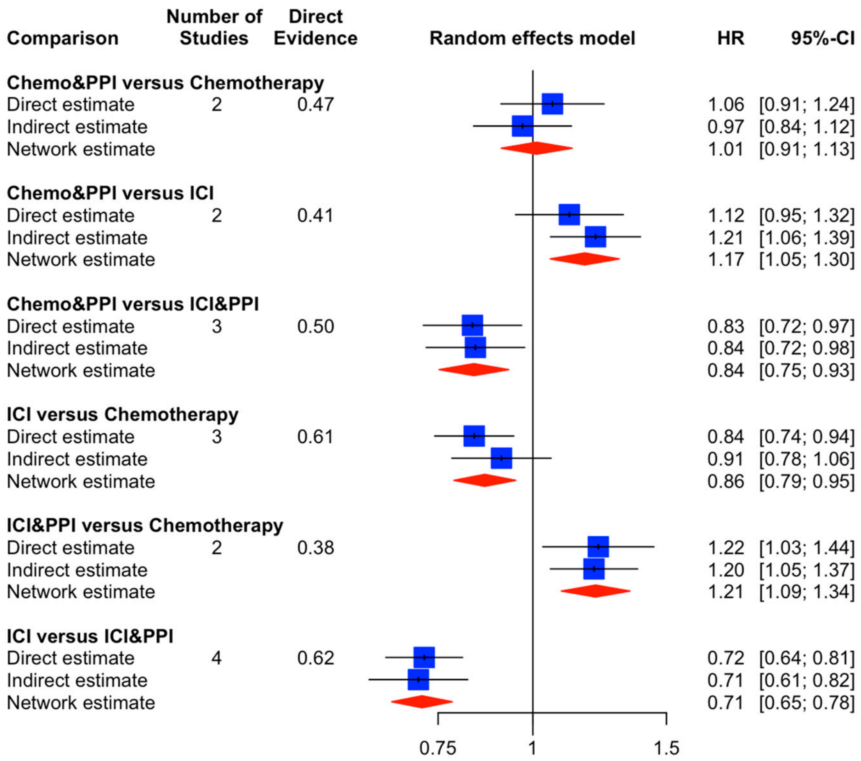

Figure S28. Forest plot of netsplitting direct and indirect network estimates for hazard ratio (HR) of progression-free survival after excluding studies of unknown PDL-1 status. No significant inconsistency was demonstrated. ICI, immune checkpoint inhibitors; PPI, proton pump inhibitors; chemo, chemotherapy

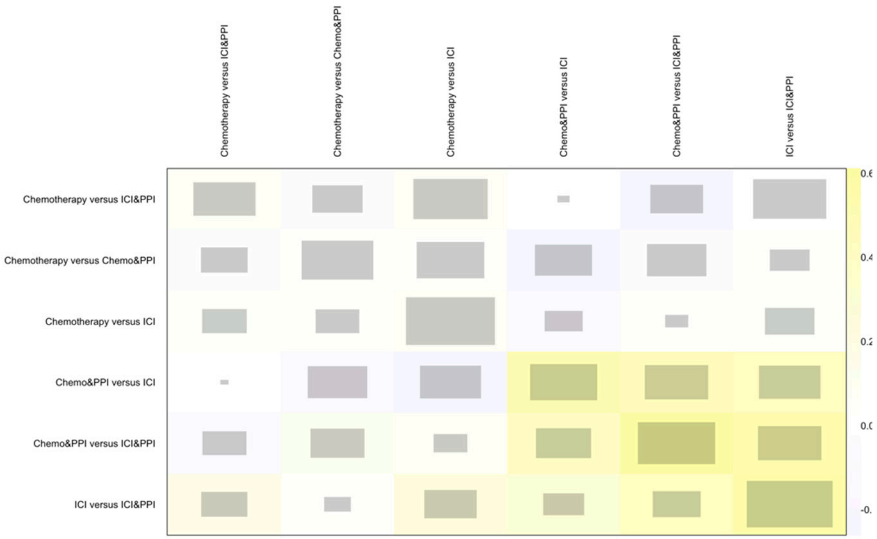

Figure S29. Netheat plot of network estimates for hazard ratio (HR) of overall survival after excluding studies of unknown PDL-1 status. The gray squares indicate the direct estimates. The colors are associated with the change in inconsistency between direct and indirect comparison. Intense color (eg, red) indicates stronger inconsistency.
No significant inconsistency was demonstrated. ICI, immune checkpoint inhibitors; PPI, proton pump inhibitors; chemo, chemotherapy

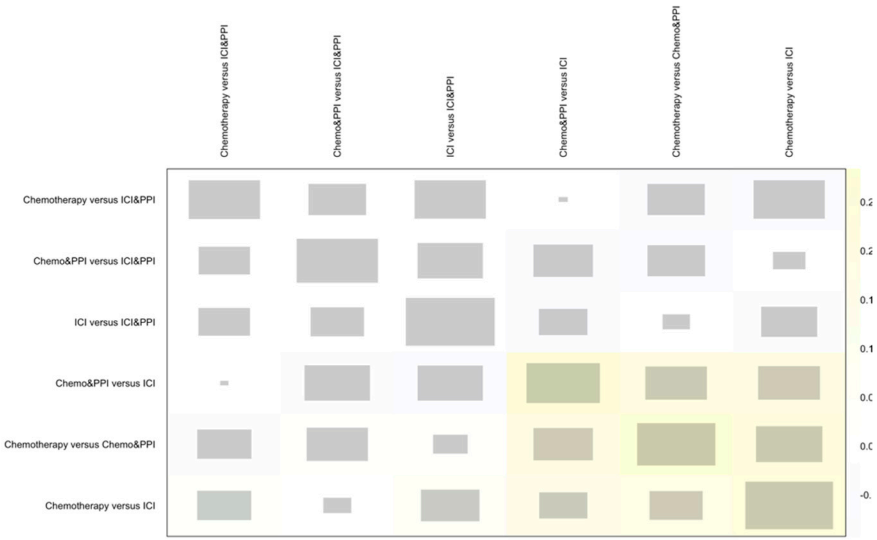

Figure S30. Forest plot of netsplitting direct and indirect network estimates for hazard ratio (HR) of progression-free survival after excluding studies of unknown PDL-1 status. No significant inconsistency was demonstrated. ICI, immune checkpoint inhibitors; PPI, proton pump inhibitors; chemo, chemotherapy

Result 7. Assessment of publication bias in pairwise meta-analysis of ICI cohorts

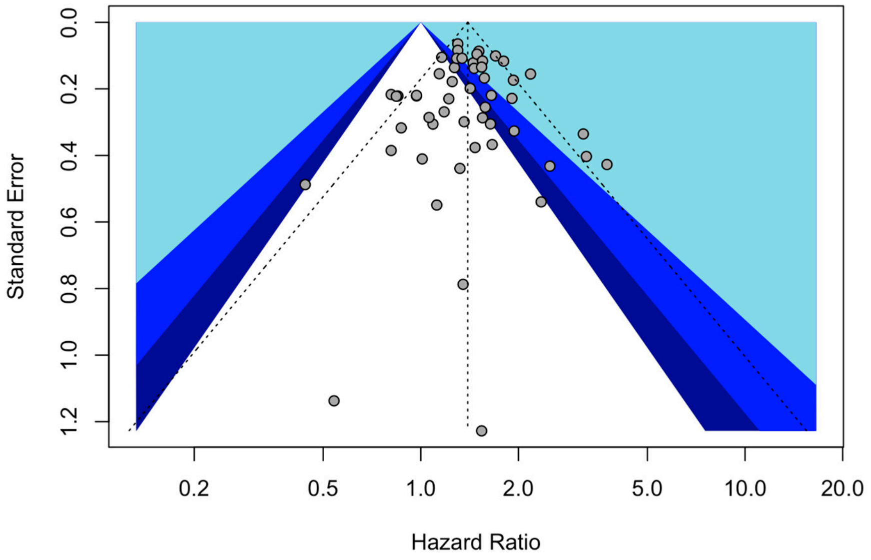

Figure S31. Assessment of publication bias of overall survival in ICI cohorts through funnel plots. Funnel plot demonstrates no significant asymmetry through visualization

Linear regression test of funnel plot asymmetry:

Test result: t = -1.31, df = 25, p-value = 0.2015
Sample estimates:

Bias	se.bias	intercept	se.intercept
-0.5310	0.4048	0.4500	0.0643

Details:
- multiplicative residual heterogeneity variance (tau^2 = 1.2824)
- predictor: standard error
- weight: inverse variance
- reference: Egger et al. (1997), BMJ

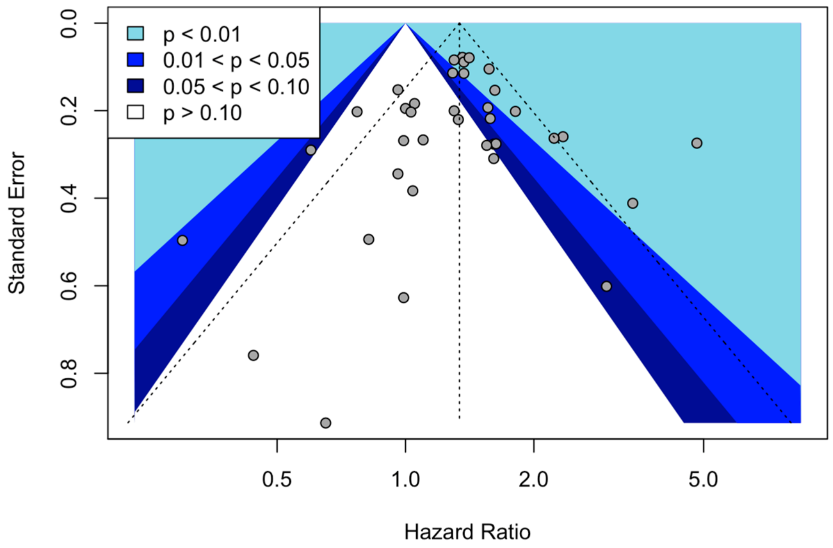

Figure S32. Assessment of publication bias of progression-free survival in ICI cohorts through funnel plots. Funnel plot demonstrates no significant asymmetry through visualization

Linear regression test of funnel plot asymmetry

Test result: t = 0.00, df = 24, p-value = 0.9969

Sample estimates:

bias	se.bias	intercept	se.intercept
0.0022	0.5672	0.3340	0.0890

Details:
- multiplicative residual heterogeneity variance (tau^2 = 2.7229)
- predictor: standard error
- weight: inverse variance
- reference: Egger et al. (1997), BMJ

Result 8. Sensitivity analysis of excluding studies subject to high risk of bias

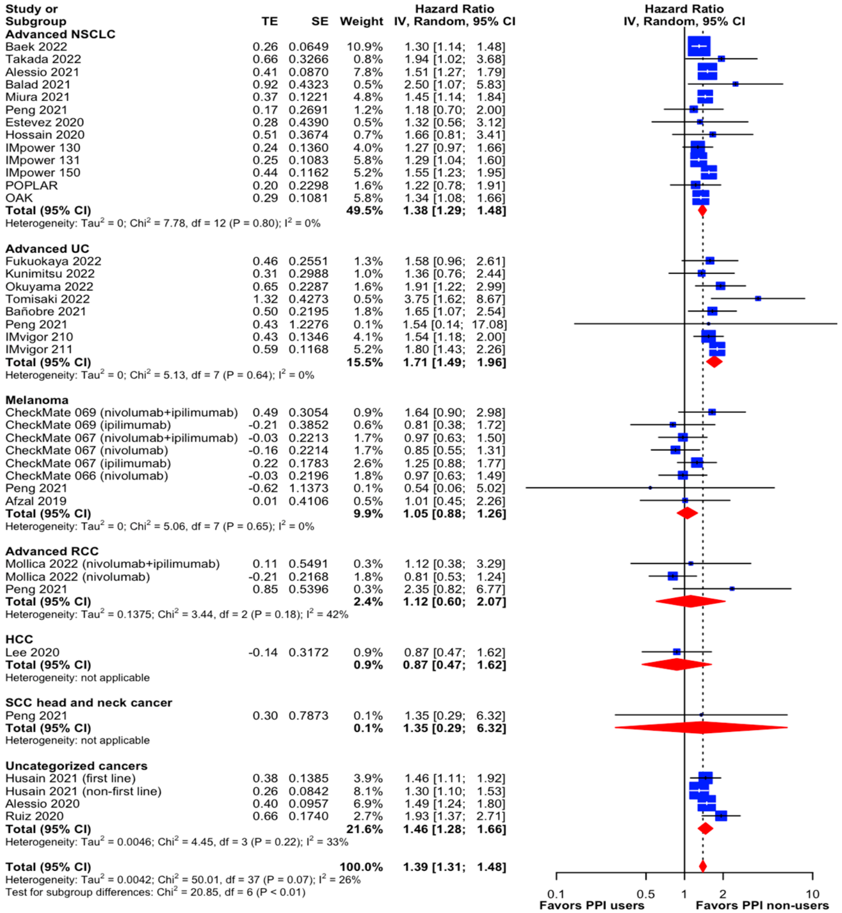

Figure S33. Forest plot of comparative overall-survival in cancer patients treated with ICI between PPI users and non-users. The size of squares is proportional to the weight of each study. Horizontal lines indicate the 95% CI of each study; diamond, the pooled estimate with 95%.
CI, confidential interval, HCC, hepatocellular carcinoma, HR, hazard ratio, NSCLC, non-small cell lung cancer, PPI, proton pump inhibitors, RCC, renal cell carcinoma, SCC, squamous cell carcinoma, UC, urothelial carcinoma

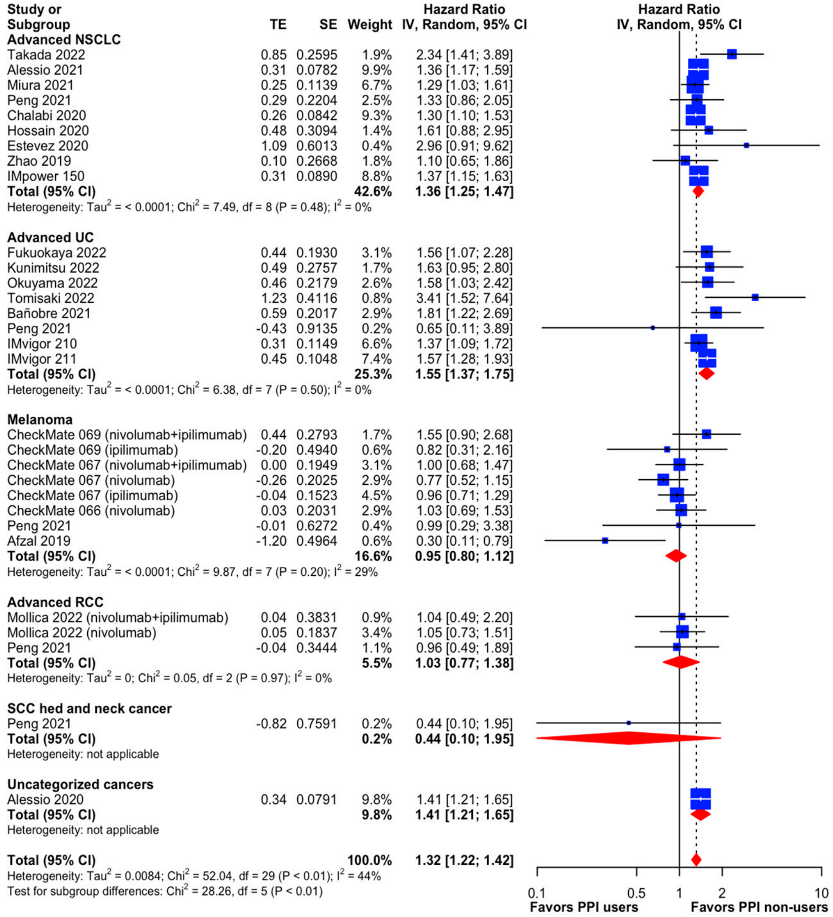

Figure S34. Forest plot of comparative progression-free in cancer patients treated with ICI between PPI users and non-users. The size of squares is proportional to the weight of each study. Horizontal lines indicate the 95% CI of each study; diamond, the pooled estimate with 95%.
CI, confidential interval, HR, hazard ratio, NSCLC, non-small cell lung cancer, PPI, proton pump inhibitors, RCC, renal cell carcinoma, SCC, squamous cell carcinoma, UC, urothelial carcinoma

Result 9. Pairwise meta-analyses of chemotherapy cohort

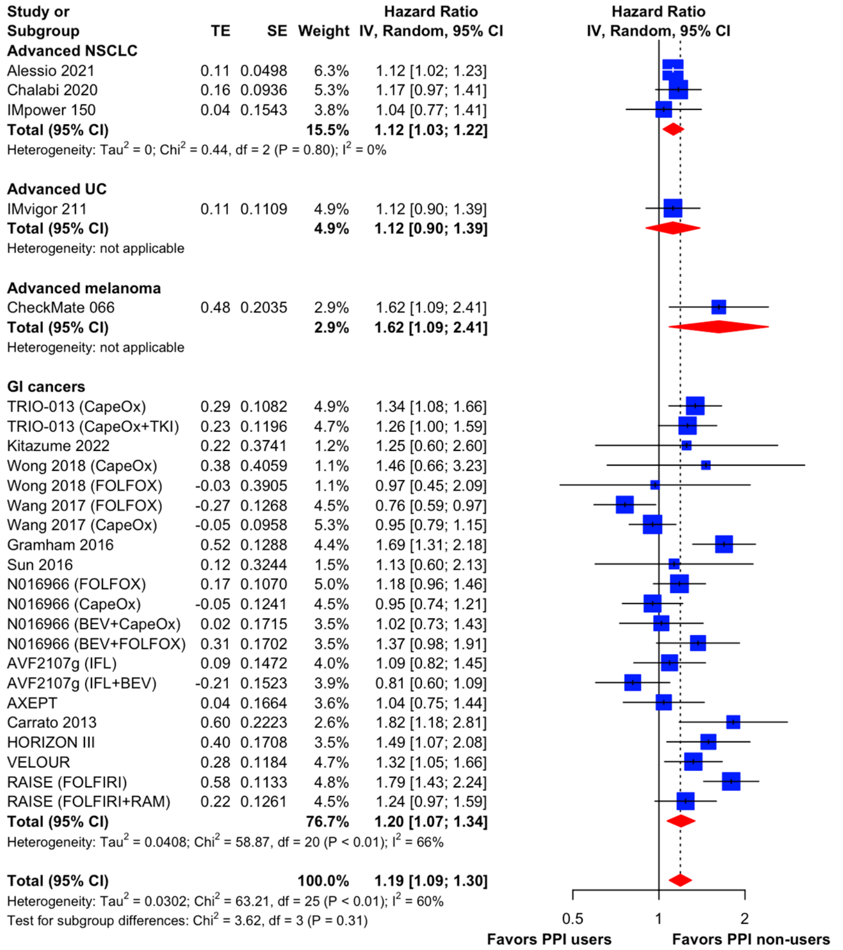

Figure S35. Forest plot of overall survival in cancer patients receiving chemotherapy with PPI users versus PPI non-users. Pooled unadjusted HRs with 95% CIs were calculated using random-effects models by adopting the restricted maximum-likelihood method as the heterogeneity estimator. NSCLC, non-small cell lung cancer; UC, urothelial carcinoma.

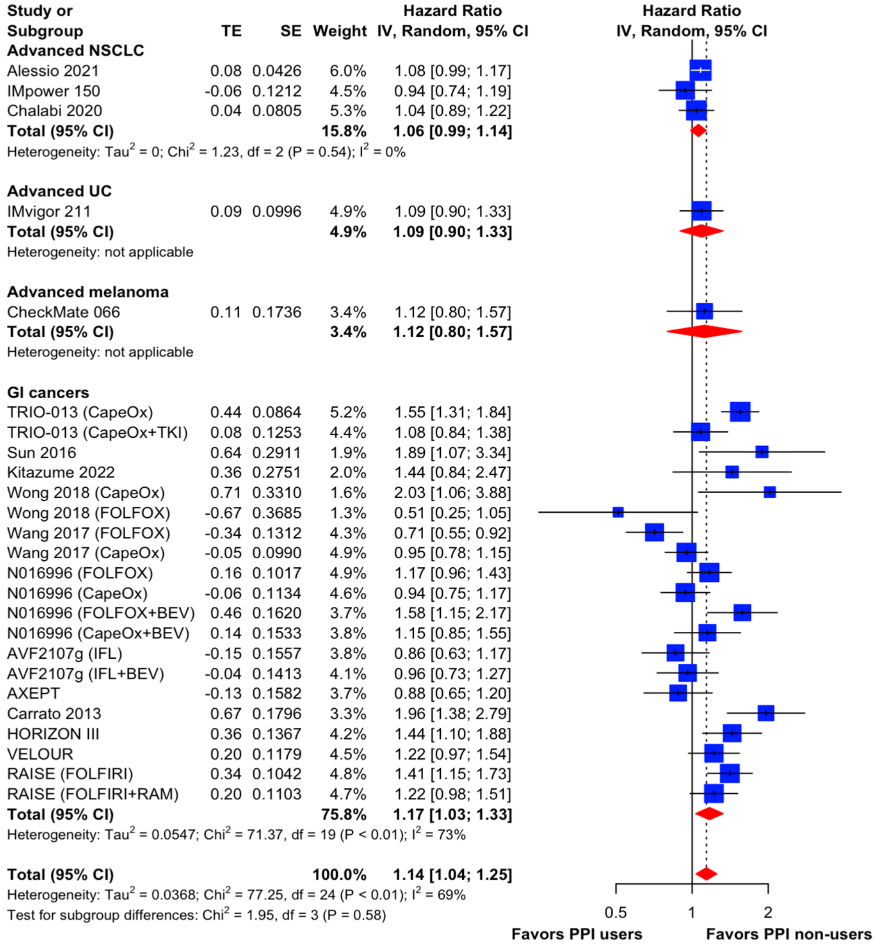

Figure S36. Forest plot of progression-free survival in cancer patients receiving chemotherapy with PPI users versus PPI non-users. Pooled unadjusted HRs with 95% CIs were calculated using random-effects models by adopting the restricted maximum-likelihood method as the heterogeneity estimator. NSCLC, non-small cell lung cancer; UC, urothelial carcinoma.

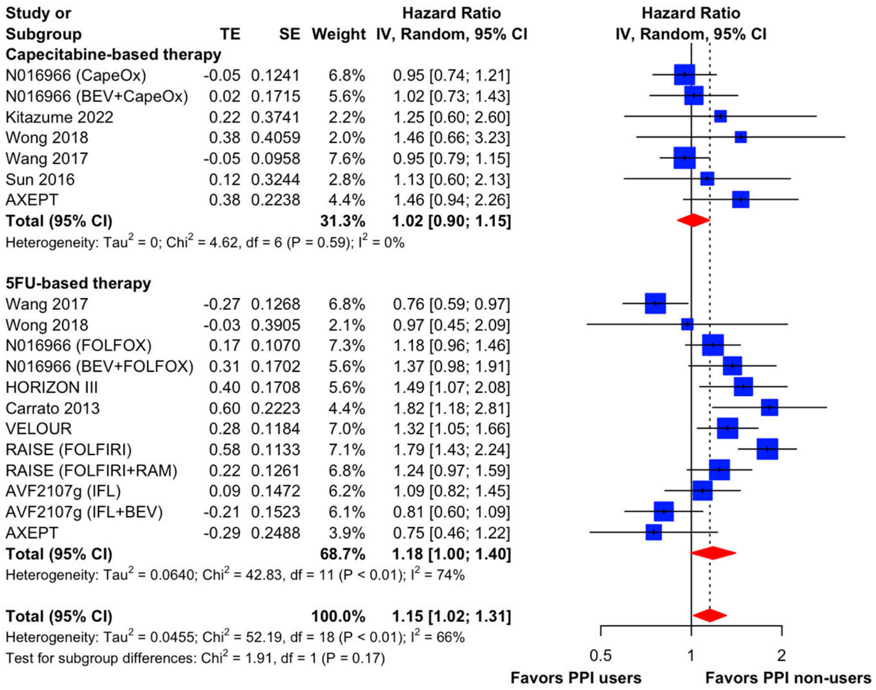

Figure S37. Forest plot of subgroup analysis regarding overall survival in colorectal cancer patients receiving chemotherapy with PPI users versus PPI non-users. Pooled unadjusted HRs with 95% CIs were calculated using random-effects models by adopting the restricted maximum-likelihood method as the heterogeneity estimator. FU, fluorouracil

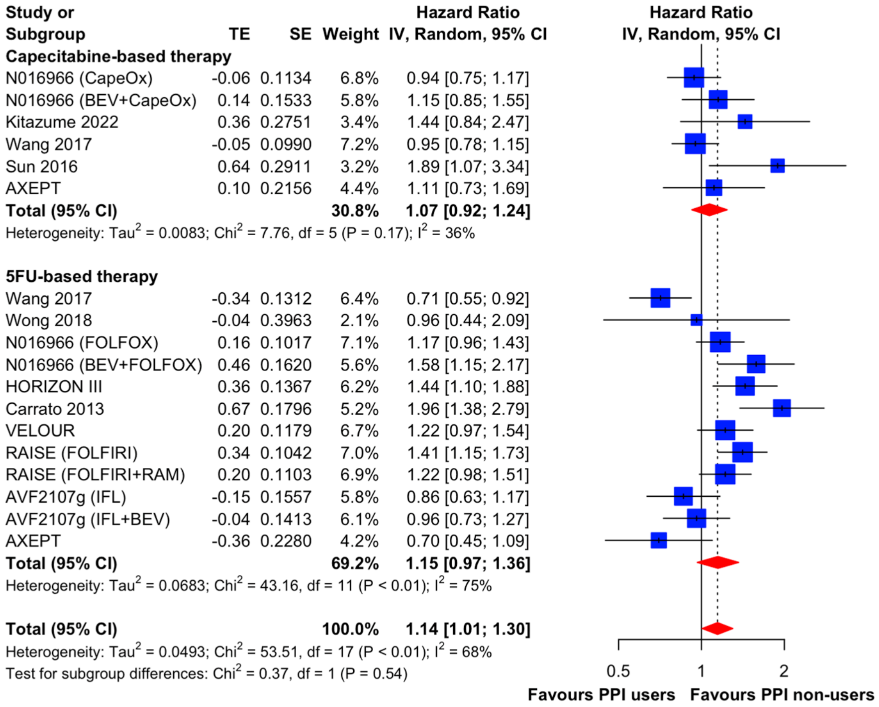

Figure S38. Forest plot of subgroup analysis regarding progression-free survival in colorectal cancer patients receiving chemotherapy with PPI users versus PPI non-users. Pooled unadjusted HRs with 95% CIs were calculated using random-effects models by adopting the restricted maximum-likelihood method as the heterogeneity estimator. FU, fluorouracil

Result 10. Pairwise meta-analyses using adjusted Hazard Ratios (HR)

Study or Subgroup	TE	SE	Weight	Hazard Ratio IV, Random, 95% CI
**Advanced NSCLC**				
Baek 2022	0.25	0.0654	9.0%	1.28 [1.13; 1.45]
Takada 2022	0.94	0.3412	1.5%	2.55 [1.31; 4.98]
Alessio 2021	0.40	0.0867	7.9%	1.49 [1.26; 1.77]
Peng 2021	0.06	0.2886	2.0%	1.06 [0.60; 1.87]
Miura 2021	0.31	0.1755	4.2%	1.36 [0.96; 1.92]
Svaton 2020	0.20	0.2669	2.3%	1.22 [0.72; 2.06]
IMpower 130	0.11	0.1397	5.4%	1.12 [0.85; 1.47]
IMpower 131	0.21	0.1136	6.5%	1.23 [0.98; 1.54]
IMpower 150	0.43	0.1217	6.2%	1.53 [1.21; 1.94]
POPLAR	0.15	0.2448	2.6%	1.16 [0.72; 1.87]
OAK	0.22	0.1093	6.7%	1.25 [1.01; 1.55]
**Total (95% CI)**			**54.5%**	**1.32 [1.23; 1.42]**
Heterogeneity: Tau^2 = < 0.0001; Chi^2 = 10.36, df = 10 (P = 0.41); I^2 = 3%				
**Advanced UC**				
Okuyama 2022	0.58	0.2786	2.2%	1.78 [1.03; 3.07]
Tomisaki 2022	1.39	0.6065	0.5%	4.00 [1.22; 13.13]
Bañobre 2021	0.49	0.2308	2.9%	1.64 [1.04; 2.58]
IMvigor 211	0.53	0.1249	6.0%	1.70 [1.33; 2.17]
IMvigor 210	0.34	0.1421	5.3%	1.40 [1.06; 1.85]
**Total (95% CI)**			**16.9%**	**1.62 [1.38; 1.90]**
Heterogeneity: Tau^2 = < 0.0001; Chi^2 = 3.54, df = 4 (P = 0.47); I^2 = 0%				
**Melanoma**				
Gaucher 2021	0.12	0.3694	1.3%	1.13 [0.55; 2.33]
**Total (95% CI)**			**1.3%**	**1.13 [0.55; 2.33]**
Heterogeneity: not applicable				
**Uncategorized cancers**				
Araujo 2021	0.55	0.1747	4.2%	1.73 [1.23; 2.44]
Gaucher 2021	-0.22	0.1499	5.0%	0.80 [0.60; 1.07]
Giordan 2021 (antibiotics-)	0.64	0.2188	3.1%	1.89 [1.23; 2.90]
Giordan 2021 (antibiotics+)	0.75	0.2219	3.1%	2.12 [1.37; 3.28]
Peng 2021	0.18	0.2264	3.0%	1.20 [0.77; 1.87]
Alessio 2020	0.23	0.0968	7.4%	1.26 [1.04; 1.52]
Santamaria 2020	-0.24	0.3472	1.5%	0.79 [0.40; 1.56]
**Total (95% CI)**			**27.2%**	**1.33 [1.00; 1.76]**
Heterogeneity: Tau^2 = 0.1025; Chi^2 = 23.18, df = 6 (P < 0.01); I^2 = 74%				
**Total (95% CI)**			**100.0%**	**1.36 [1.25; 1.49]**
Heterogeneity: Tau^2 = 0.0190; Chi^2 = 43.13, df = 23 (P < 0.01); I^2 = 47%				
Test for subgroup differences: Chi^2 = 5.31, df = 3 (P = 0.15)				

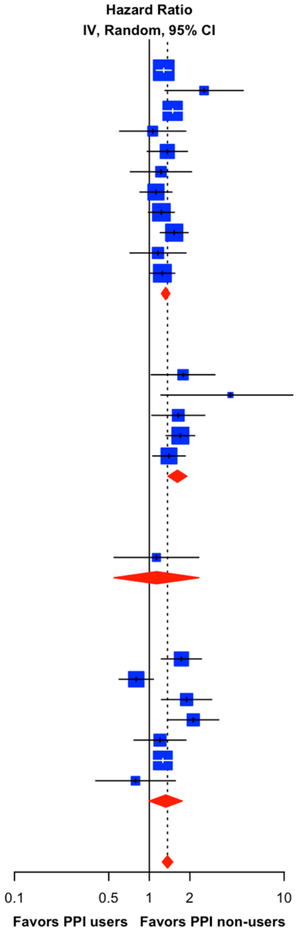

Figure S39. Forest plot of adjusted OS in cancer patients receiving ICI with PPI users versus PPI non-users. Pooled adjusted HRs with 95% CIs were calculated using random-effects models by adopting the restricted maximum-likelihood method as the heterogeneity estimator. OS, overall survival; NSCLC, non-small cell lung cancer; UC, urothelial carcinoma.

Study or Subgroup	TE	SE	Weight	Hazard Ratio IV, Random, 95% CI
**Advanced NSCLC**				
Takada 2022	1.42	0.3024	4.4%	4.12 [2.28; 7.45]
Alessio 2021	0.28	0.0790	7.1%	1.32 [1.13; 1.54]
Miura 2021	0.16	0.1506	6.3%	1.17 [0.87; 1.57]
Peng 2021	0.24	0.2210	5.4%	1.27 [0.82; 1.96]
IMpower 150	0.29	0.0926	6.9%	1.34 [1.12; 1.61]
Estevez 2020	1.07	0.6109	2.0%	2.91 [0.88; 9.64]
Svaton 2020	0.31	0.2141	5.5%	1.36 [0.89; 2.07]
**Total (95% CI)**			**37.7%**	**1.51 [1.15; 1.99]**
Heterogeneity: Tau^2 = 0.0904; Chi^2 = 16.25, df = 6 (P = 0.01); I^2 = 63%				
**Advanced UC**				
Okuyama 2022	0.54	0.2427	5.1%	1.72 [1.07; 2.77]
Tomisaki 2022	1.21	0.5369	2.4%	3.36 [1.17; 9.62]
Bañobre 2021	0.66	0.2371	5.2%	1.94 [1.22; 3.09]
IMvigor 210	0.26	0.1211	6.7%	1.30 [1.03; 1.65]
IMvigor 211	0.40	0.1126	6.8%	1.49 [1.20; 1.86]
**Total (95% CI)**			**26.2%**	**1.50 [1.29; 1.74]**
Heterogeneity: Tau^2 = 0.0008; Chi^2 = 5.15, df = 4 (P = 0.27); I^2 = 22%				
**Uncategorized cancers**				
Araujo 2021	0.86	0.1768	6.0%	2.36 [1.67; 3.34]
Giordan 2021 (antibiotics-)	0.41	0.1565	6.3%	1.51 [1.11; 2.05]
Giordan 2021 (antibiotics+)	1.29	0.1442	6.4%	3.65 [2.75; 4.84]
Peng 2021	-0.11	0.1768	6.0%	0.90 [0.64; 1.27]
Alessio 2020	0.23	0.0828	7.0%	1.26 [1.07; 1.48]
Santamaria 2020	-0.29	0.2960	4.5%	0.75 [0.42; 1.34]
**Total (95% CI)**			**36.2%**	**1.52 [0.95; 2.43]**
Heterogeneity: Tau^2 = 0.3084; Chi^2 = 62.67, df = 5 (P < 0.01); I^2 = 92%				
**Total (95% CI)**			**100.0%**	**1.58 [1.30; 1.92]**
Heterogeneity: Tau^2 = 0.1352; Chi^2 = 86.92, df = 17 (P < 0.01); I^2 = 80%				
Test for subgroup differences: Chi^2 = 0.01, df = 2 (P = 1.00)				

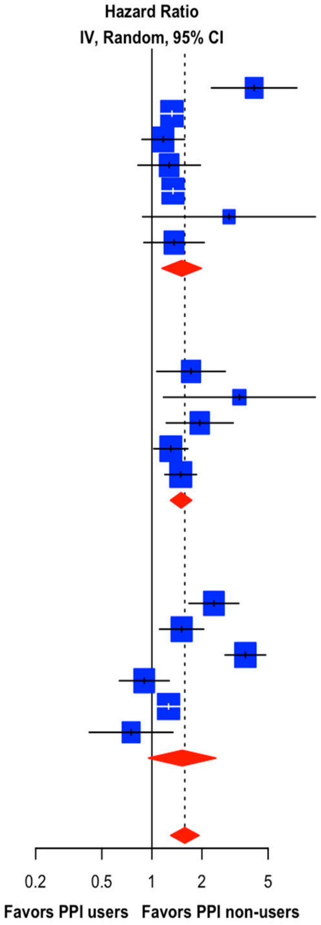

Figure S40. Forest plot of adjusted PFS in cancer patients receiving ICI with PPI users versus PPI non-users. Pooled adjusted HRs with 95% CIs were calculated using random-effects models by adopting the restricted maximum-likelihood method as the heterogeneity estimator. PFS, progression-free survival; NSCLC, non-small cell lung cancer; UC, urothelial carcinoma

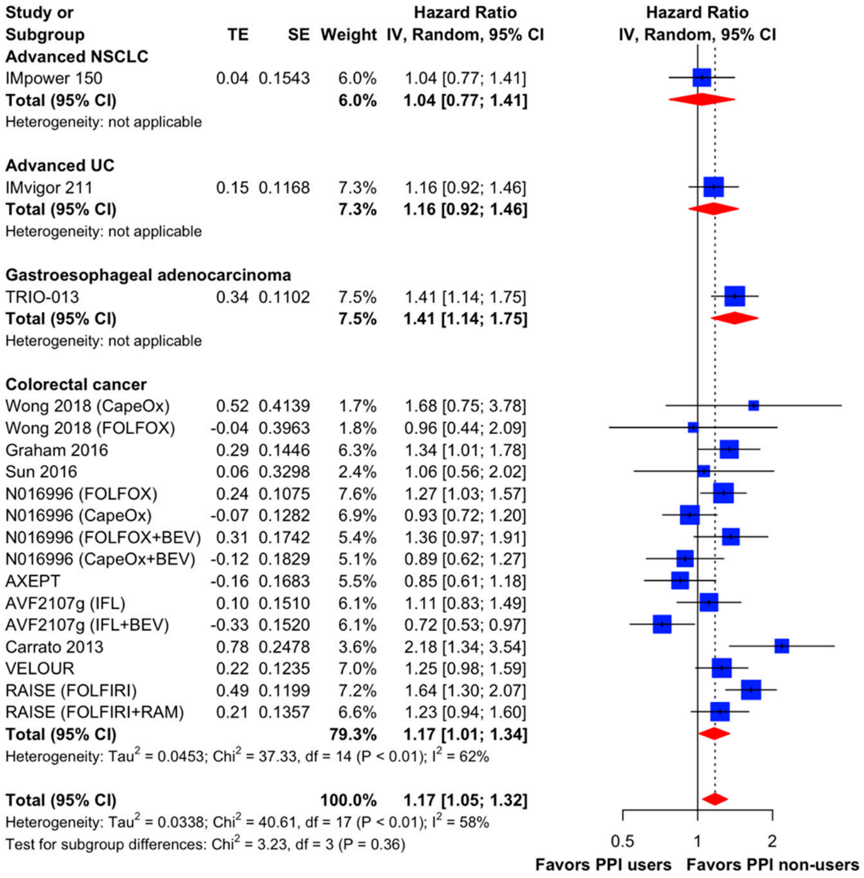

Figure S41. Forest plot of adjusted overall survival in cancer patients receiving chemotherapy with PPI users versus PPI non-users. Pooled adjusted HRs with 95% CIs were calculated using random-effects models by adopting the restricted maximum-likelihood method as the heterogeneity estimator. UC, urothelial carcinoma; mCRC, metastatic colorectal cancer.

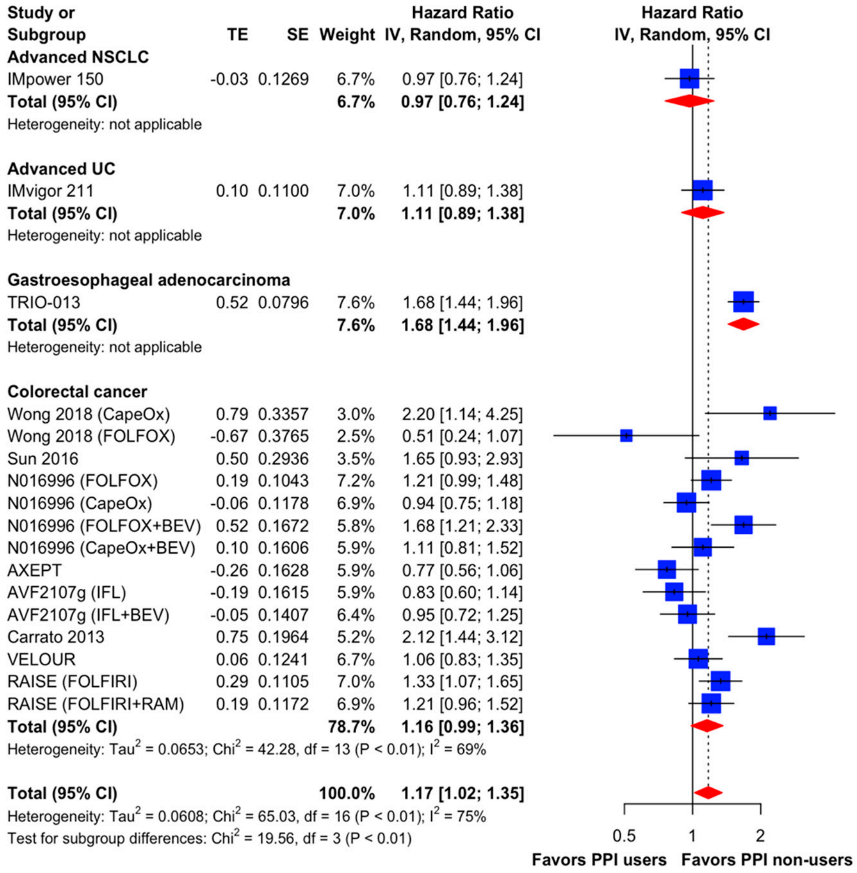

Figure S42. Forest plot of adjusted progression-free survival in cancer patients receiving chemotherapy with PPI users versus PPI non-users. Pooled adjusted HRs with 95% CIs were calculated using random-effects models by adopting the restricted maximum-likelihood method as the heterogeneity estimator. UC, urothelial carcinoma; mCRC, metastatic colorectal cancer.

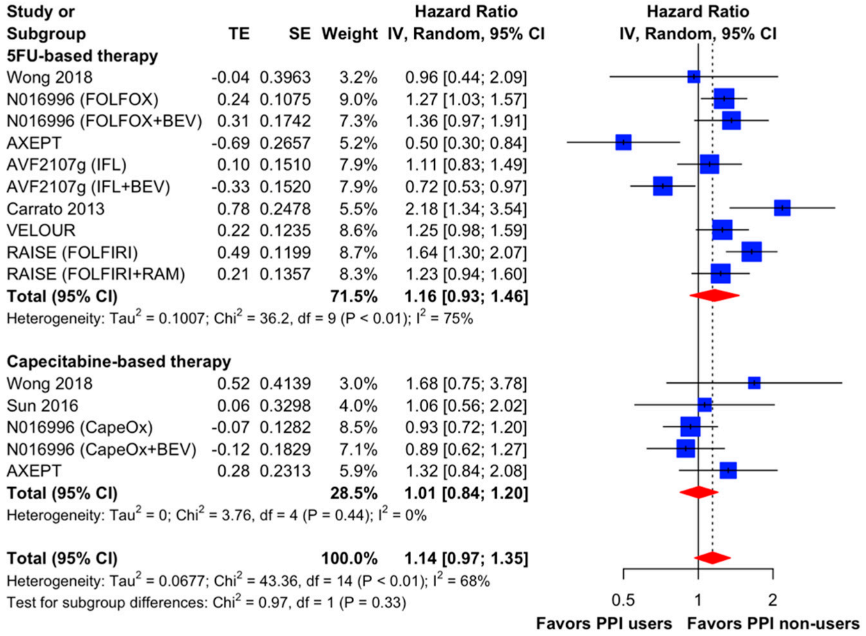

Figure S43. Forest plot of subgroup analysis regarding overall survival in colorectal cancer patients receiving chemotherapy with PPI users versus PPI non-users. Pooled adjusted HRs with 95% CIs were calculated using random-effects models by adopting the restricted maximum-likelihood method as the heterogeneity estimator. FU, fluorouracil

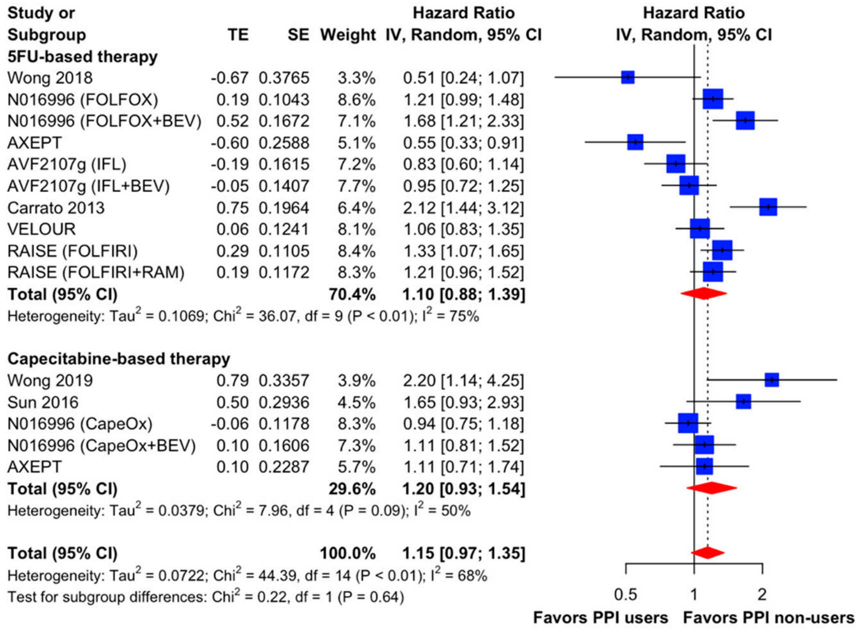

Figure 44. Forest plot of subgroup analysis regarding progression-free survival in colorectal cancer patients receiving chemotherapy with PPI users versus PPI non-users. Pooled adjusted HRs with 95% CIs were calculated using random-effects models by adopting the restricted maximum-likelihood method as the heterogeneity estimator. FU, fluorouracil

Table S1. Eligibility criteria of trial patients included in NMA		
Trial	Inclusion criteria	Exclusion criteria
IMpower 130	1. Age>18 histologically or cytologically confirmed stage IV squamous NSCLC.<br>2. ECOG-PS 0-1<br>3. Patients received no previous chemotherapy for stage IV squamous NSCLC<br>4. Patient with sensitizing mutation in EGFR gene or ALK fusion oncogene must have had disease progression or intolerance to treatment with at least one tyrosine kinase inhibitor (discontinue >7 days before randomization).<br>5. Patient with unknown EGFR or ALK status required test result at screening.<br>6. Previous neoadjuvant or adjuvant chemotherapy, radiotherapy, or chemoradiotherapy with curative intent for non-metastatic disease were permitted if treatment-free interval >6 months.	1. CNS metastasis, spinal cord compression or leptomeningeal disease.<br>2. History of autoimmune disease<br>3. Patient with malignancy other than NSCLC within the 5 years before randomization.<br>4. Patient with history of interstitial lung disease.<br>5. Patient treated with CD137 agonist or immune checkpoint blockade such as anti-PD-1 therapeutic antibodies or anti-PD-L1 therapeutic antibodies
IMpower 131	1. Patient had histologically or cytologically confirmed stage IV squamous NSCLC.<br>2. Patient had not yet received chemotherapy for stage IV squamous NSCLC<br>3. Baseline ECOG-PS 0- 1<br>4. Patient had tumor tissue available for central PD-L1 testing<br>5. Patient known to have EGFR mutation or ALK fusion oncogene had to have experienced disease progression on or intolerance to one or more approved tyrosine kinase or ALK inhibitor, respectively; testing for EGFR and ALK status was not mandated.	1. Patient with active or untreated CNS metastasis<br>2. History of autoimmune disease<br>3. Previous immune checkpoint blockade therapies with the exception of an anti-CTLA-4 therapy, provided the last dose was given≥ 6 weeks before randomization<br>4. Patient with systemic immunosuppressive medication less than 2 weeks before randomization.
IMpower 150	1. Patient had stage IV or recurrent metastatic non-squamous NSCLC<br>2. Patient had not previously received chemotherapy<br>3. Baseline ECOG-PS 0-1<br>4. Patient with any PD-L1 immunohistochemistry status<br>5. Patient with EGFR or ALK genomic alteration if they had had disease progression with or unacceptable side effects from treatment with at least one	1. Patient had untreated metastasis of the CNS<br>2. Patient had autoimmune disease<br>3. Patient had received previous immunotherapy or anti-CTLA-4 therapy within 6 weeks before randomization or systemic immunosuppressive medications within 2 weeks before randomization.

	tyrosine kinase inhibitor.<br>6. Patient received previous adjuvant or neoadjuvant chemotherapy if the last treatment was at least 6 months before randomization	
IMvigor 210	1. Age ≥ 18, with metastatic urothelial carcinoma and measurable disease at baseline as per Response Evaluation Criteria In Solid Tumors version 1.1 (RECIST v1.1);<br>2. ECOG-PS 0-1;<br>3. Cohort 1: patients without previous treatment for inoperable, locally advanced or metastatic urothelial cancer;<br>Cohort 2: patients previously treated with platinum-based chemotherapy;<br>4. Cisplatin ineligible as per at least one of the following:<br>glomerular filtration rate more than 30 mL/min and less than 60 mL/min, grade 2 or higher hearing loss or peripheral neuropathy;<br>5. A tumor sample available for PD-L1 testing;<br>6. Neo-adjuvant or adjuvant chemotherapy or radiation was permitted if more than 12 months had elapsed between treatment and recurrence;<br>7. Any approved anti-cancer therapy was permitted if completed >3 weeks prior to initiation of study treatment	1. Pregnant and lactating women<br>2. Known CNS metastases or leptomeningeal diseases<br>3. History of autoimmune disease<br>4. Cardiovascular disease:<br>congestive heart NYHA Class II or above, unstable angina, myocardial infarction within the previous 3 months, or unstable arrhythmias<br>5. Uncontrolled pleural effusion, pericardial effusion, or ascites requiring recurrent drainage procedures (once monthly or more frequently)<br>6. Prior treatment with CD137 agonists or immune checkpoint blockade therapies, including anti-CTLA-4, anti-PD-1, and anti-PD-L1 therapeutic antibodies
IMvigor 211	1. Age ≥ 18, with metastatic urothelial carcinoma and measurable disease at baseline as per RECIST v1.1;<br>2. ECOG-PS 0-1;<br>3. An evaluable sample for PD-L1 testing;<br>4. Patients had received no more than two previous lines of therapy and had progressed during or following one or more platinum-containing regimens (or neoadjuvant or adjuvant therapy with progression within 12 months).<br>5. A predominance of transitional histology was required	1. Patients with previous autoimmune disease;<br>2. Who had received therapies targeting CD137, CTLA4, or PD-L1–PD-1;<br>3. Known CNS metastases;<br>4. Inadequate renal or liver function.
POPLAR	1. Age ≥ 18, pathohistologically documented locally advanced or metastatic NSCLC with at least one measurable lesion as defined by RECIST criteria;	1. Known CNS metastases;<br>2. History of pneumonitis, autoimmune or chronic viral diseases;

	2. ECOG-PS 0-1;<br>3. Provided tumor specimens for central PD-L1 testing on formalin-fixed paraffin-embedded (FFPE) sections before enrolment;<br>4. Adequate hematological and end-organ function;	3. Previous treatment with docetaxel, CD137 agonists, antiCTLA4, anti-PD-L1, or anti-PD-1 therapeutic antibodies, or PD-L1–PD-1 pathway-targeting agents.
OAK	1. Age ≥ 18, pathohistologically documented locally advanced or metastatic NSCLC with at least one measurable lesion as defined by RECIST criteria;<br>2. ECOG-PS 0-1, with a life expectancy of longer than 3months;<br>3. Disease progression during or following treatment with a prior platinum-containing regimen;	1. Pregnant or breast-feeding women;<br>2. Known CNS metastases;<br>3. Cardiovascular disease:<br>CHF NYHA (Class II or greater), myocardial infarction within 3 months prior to randomisation, unstable arrhythmias or unstable angina<br>4. Uncontrolled concomitant diseases:<br>cirrhosis, uncontrolled major seizure disorder or SVC syndrome<br>5. Severe infections within 4 weeks prior to randomisation including but not limited to hospitalization for complications of infection, bacteremia or severe pneumonia<br>6. Received therapeutic oral or intravenous antibiotics within 2 weeks prior to randomisation<br>7. Major surgical procedure within 4 weeks prior to randomisation or anticipation of need for a major surgical procedure during the course of the study other than for diagnosis<br>8. Previous treatment with docetaxel, CD137 agonists, antiCTLA4, anti-PD-L1, or anti-PD-1 therapeutic antibodies, or PD-L1–PD-1 pathway-targeting agents.
CheckMate 066	1. Patients had confirmed, unresectable, previously untreated stage III or IV melanoma without a BRAF mutation.<br>2. Age> 18<br>3. ECOG-PS between 0-1	1. Active brain metastasis<br>2. Uveal melanoma<br>3. Severe autoimmune disease

	4. Availability of tumor tissue from a metastatic or unresectable site for PD-L1 biomarker analysis.<br>5. Patients who had received adjuvant therapy previously were included.	
CheckMate 067	1. Patients with previously untreated, histologically confirmed stage III or stage IV melanoma, with known BRAF V600 mutation status.<br>2. Age>18<br>3. ECOG between 0-1<br>4. Availability of tumor tissue from a metastatic or unresectable site for PD-L1 biomarker analysis.	1. Active brain metastasis<br>2. Ocular melanoma<br>3. Autoimmune disease<br>4. ECOG-PS =2
CheckMate 069	1. Patients with previously untreated, histologically confirmed stage III or stage IV melanoma, with known BRAF V600 mutation status.<br>2. Age> 18<br>3. ECOG between 0-1<br>4. Availability of tumor tissue from a metastatic or unresectable site for PD-L1 biomarker analysis.<br>5. Any prior radiotherapy must have been completed at least 2 weeks before study drug administration.<br>6. Previous adjuvant or neoadjuvant treatment for melanoma was allowed if it had been completed at least 6 weeks before the date of first dose, and all related adverse events either returned to baseline or stabilized.	1. Patients are pregnant or breastfeeding<br>2. Active brain metastasis<br>3. Ocular melanoma<br>4. Patients who had received previous systemic anticancer therapy for unresectable or metastatic melanoma.
AVF2107	6. Age>18, with histologically confirmed metastatic colorectal cancer (MCRC), bidimensional measurable lesions;<br>7. ECOG-PS 0-1, with a life expectancy of longer than 3 months;<br>8. Adequate hematologic, hepatic, and renal function (including urinary excretion of no more than 500 mg of protein per day).<br>9. Prior radiotherapy for MCRC was permitted if completed > 2 weeks before and random assignment	5. Pregnant or breast-feeding women;<br>6. Known CNS metastases;<br>7. Cardiovascular disease;<br>8. Clinically detectable ascites;<br>9. Regular use of aspirin (more than 325 mg per day) or other nonsteroidal anti-inflammatory agents;

	10. Major surgery was permitted if completed > 4 weeks before and random assignment	10. Preexisting bleeding diatheses or coagulopathy or the need for full-dose anticoagulation;<br>11. Prior chemotherapy or biologic therapy for metastatic disease
N016966	1. Age>18, with histologically confirmed MCRC, one or more uni-dimensionally measurable lesions;<br>2. ECOG-PS 0-1, with a life expectancy of longer than 3months;<br>3. No prior systemic therapy for MCRC;<br>4. Radiotherapy or surgery for MCRC was permitted if completed > 4 weeks before random assignment	1. Pregnant or breast-feeding women;<br>2. Known CNS metastases;<br>3. Cardiovascular disease;<br>4. Clinically detectable ascites;<br>5. Use of full-dose anticoagulants or thrombolytics;<br>6. Serious nonhealing wound, ulcer, or bone fracture;<br>7. Clinically significant bleeding diathesis or coagulopathy; and<br>8. Proteinuria > 500 mg/24 hours.
Carrato 2013	1. Age ≥ 18, with histologically confirmed MCRC, with at least one measurable lesion as defined by RECIST criteria;<br>2. ECOG-PS 0-1;<br>3. Radiotherapy was permitted if full-field is completed > 4 weeks or limited field is completed > 2 weeks before random assignment<br>4. Major surgical procedure, open biopsy or significant traumatic injury was permitted if >4 weeks before randomization;	1. Pregnant or lactating women;<br>2. Prior treatment with a VEGF, VEGFR or RTK inhibitor;<br>3. Cardiovascular diseases:<br>poorly controlled hypertension, coronary artery diseases, congestive heart failure, ongoing arrythmia, cerebrovascular accident, thromboembolism;<br>4. GI comorbidities:<br>peptic ulcer disease, infectious or inflammatory bowel disease, diverticulitis, unresolved bowel obstruction or chronic diarrhea, recent history of abdominal fistula, gastrointestinal perforation, or intra-abdominal abscess within 6 months prior to study enrollment<br>5. Known CNS metastases;<br>6. Use of full-dose anticoagulants;<br>7. History of clinically significant bleeding within the past 6 months, including gross hemoptysis or hematuria, or underlying coagulopathy;<br>8. Active infection, or on antiretroviral therapy for HIV disease.
VELOUR	1. Age ≥ 18, with pathohistologically confirmed MCRC<br>2. ECOG PS 0-2;.	1. Pregnant or lactating women;<br>2. Known prior malignancies or CNS metastases;

	3. Patients have documented progression while on or after completion of a single prior oxaliplatin-containing regimen;<br>4. Prior bevacizumab was permitted, but not prior irinotecan.<br>5. Major surgery was permitted if completed > 4 weeks before randomization	3. Severe acute or chronic medical condition that may have impaired the ability to participate in the study or interfered with the interpretation of results;<br>4. Poorly controlled hypertension or thromboembolism.
RAISE	1. Age> 18, with pathologically confirmed MCRC, known KRAS exon 2 mutation (mutant or wild-type);<br>2. ECOG-PS 0-1;<br>3. Eligible patients had disease progression during or within 6 months of the last dose of first-line combination therapy with bevacizumab, oxaliplatin, and a fluoropyrimidine, and had received at least one cycle of the triplet therapy<br>4. Prior bevacizumab was permitted if completed > 4 weeks before random assignment<br>5. Prior chemotherapy was permitted if completed > 3 weeks before randomization	1. Known CNS metastases;<br>2. Poorly controlled hypertension;<br>3. Any thromboembolism within 12 months before randomisation or during first-line therapy;<br>4. Grade 3–4 bleeding event, Grade 3 proteinuria, or bowel perforation during first-line therapy;<br>5. Grade 3–4 bleeding within 3 months before randomisation

Table S2. Details of uncategorized cancers

Study	Sample size, n	Cancer types						PDL-1 expression		
		NSCLC, n (%)	Melanoma, n (%)	RCC, n (%)	UC, n (%)	HNSCC, n (%)	Others, n (%)	TC/IC0, n (%)	TC/IC1/2, n (%)	TC/IC3, n (%)
Araujo 2021	216	39 (18.1)	33 (15.2)	0	0	N/A	N/A	N/A	N/A	N/A
Buti 2021	217	152 (70.0)	32 (14.7)	20 (9.2)	N/A	N/A	13 (5.9)	N/A	N/A	N/A
Gaucher 2021	370	166 (44.6)	110 (29.6)	27 (7.3)		N/A	69 (18.5)	N/A	N/A	N/A
Giordan 2021	212	65 (30.7)	76 (35.9)	33 (15.6)	0	38 (17.9)	0	13 (29.6)	10 (22.7)	21 (47.7)
Husain 2021	1091	100		N/A	N/A	N/A	N/A	N/A	N/A	N/A
Peng 2021	233	117 (50.2)	35 (15.0)	52 (22.3)	8 (3.4)	21 (9.0)	0	N/A	N/A	N/A
Alessio 2020	1012	528 (52.2)	263 (26.0)	185 (18.3)	N/A	N/A	36 (3.6)	N/A	N/A	N/A
Kostine 2021	635	150 (23.6)	293 (46.1)	83 (13.0)	16 (2.5)	48 (7.6)	45 (7.1)	N/A	N/A	N/A
Ruiz 2020	635	120 (47.4)	96 (38.0)	N/A	N/A	N/A	37 (14.6)	N/A	N/A	N/A
Santamaria 2019	102	56 (54.9)	10 (9.8)	12 (11.8)	11 (10.8)	N/A	13 (12.7)	N/A	N/A	N/A

Table S3. Effect modifiers across the network

	Sample size		Age (median, IQR)		Male, n (%)		ECOG 0, n (%)		ECOG 1, n (%)		TC or IC 0, n (%)		TC or IC 1/2, n (%)		TC or IC 3, n (%)	
	PPI +	PPI-	PPI +	PPI-	PPI +	PPI-	PPI +	PPI-	PPI +	PPI-	PPI +	PPI-	PPI +	PPI-	PPI +	PPI-
Overall (RR or WMD, 95% CI)			*0.89 (-0.02 to 1.81)*		*0.96 (0.90 to 1.02]*		*1.03 (0.72 to 1.48)*		*1.13 (0.90 to 1.41)*		*0.98 (0.93 to 1.04)*		*0.98 (0.92 to 1.04)*		*1.11 (0.96 to 1.27)*	
Hopkins 2022	1225	3223	65 (59 - 71)	63 (57 - 69)	801 (65.4)	2095 (65)	416 (34)	1279 (39.7)	805 (65.7)	1946 (60.4)	565 (46.1)	1503 (46.6)	440 (35.9)	1213 (37.6)	216 (17.6)	509 (15.8)
Chalabi 2020	234	523	NA	NA	146 (62.4)	325 (62.1)	61 (26.1)	206 (39.4)	172 (73.5)	316 (60.4)	90 (38.5)	221 (42.3)	142 (60.7) †		298 (57.0) †	
Hopkins 2020	471	889	67 (61 – 73)	67 (60 – 73)	359 (76)	696 (78)	167 (35.4)	420 (47.2)	295 (62.6)	454 (51.0)	146 (31.0)	275 (31.0)	309 (65.6)	582 (65.5)	16 (3.4)	32 (3.6)
Chalabi 2020	260	495	NA	NA	178 (68.5)	277 (56.0)	97 (37.3)	182 (36.8)	163 (62.7)	312 (63.0)	108 (41.5)	204 (41.2)	151 (58.1) †		288 (58.2) †	
Chu 2017	119	155	58	59	77 (64.7)	123 (79.4)	36 (30.3)	39 (25.2)	72 (60.5)	101 (65.2)	NA	NA	NA	NA	NA	NA
Chu 2017 (2)	110	161	60	61	82 (74.5)	124 (77.0)	35 (31.8)	56 (34.8)	65 (59.1)	94 (58.4)	NA	NA	NA	NA	NA	NA
Kim 2021	49	433	60 (48–69)	59 (51–66)	26 (53.1)	262 (60.5)	NA	NA	NA	NA	NA	NA	NA	NA	NA	NA
AVF2107g	159	654	59 (52-65)	60 (52-69)	98 (62)	387 (59)	84 (54)	376 (57)	75 (47)	277 (42)	NA	NA	NA	NA	NA	NA
N016966	327	1792	62 (54-69)	61 (53-67)	145 (60)	1062 (59)	122 (50)	1,023 (57)	121 (50)	762 (43)	NA	NA	NA	NA	NA	NA
Carrato 2013	43	336	59 (52-66)	58 (51-65)	25 (58)	176 (52)	21 (49)	126 (38)	13 (30)	162 (48)	NA	NA	NA	NA	NA	NA
VELOUR	111	494	61 (55-70)	61 (54-68)	59 (52)	291 (59)	57 (51)	291 (59)	54 (49)	203 (41)	NA	NA	NA	NA	NA	NA
RAISE	258	814	63 (54-69)	61 (53-67)	141 (55)	474 (58)	105 (41)	433 (53)	152 (59)	378 (46)	NA	NA	NA	NA	NA	NA
HORIZON III	88	602	NA	NA	47 (53)	353 (53)	48 (55)	343 (57)	40 (45)	257 (43)	NA	NA	NA	NA	NA	NA

Note: IQR, interquartile range; RR, risk ratio; WMD, weight mean difference; PPI, proton pump inhibitors; ECOG, Eastern Cooperative Oncology Group; NA, not available;
PDL-1 expression level: Tumour cell (TC) or tumour-infiltrating immune cell (IC) group levels (TC PDL-L1 expression: [TC0] <1%, [TC1/2] ≥1% to <50%, [TC3] ≥50%; IC PDL-L1 expression [IC0] <1%, [IC1/2] ≥1% to <50%, [IC3] ≥50%)
† PDL-1 expression data only available for TC or IC 1/2/3

Table S4. Details of proton pump inhibitors						
Included studies	PPI users, n	Omeprazole, n (%)	Pantoprazole, n (%)	Esomeprazole, n (%)	Lansoprazole, n (%)	Rabeprazole, n (%)
IMpower130	204	NA	NA	NA	NA	NA
IMpower131	259	NA	NA	NA	NA	NA
IMpower150	441	172 (39.0)	161 (36.5)	65 (14.7)	33 (7.5)	9 (2.0)
Chalabi 2020	494	219 (44.3)	151 (30.6)	73 (14.8)	66 (13.4)	8 (1.6)
Baek 2021	823	NA	NA	NA	NA	NA
Takada 2022	37	NA	NA	NA	NA	NA
Alessio 2021	474	NA	NA	NA	NA	NA
Balado 2021	26	NA	NA	NA	NA	NA
Muira 2021	163	2 (1.2)	3 (1.8)	21 (12.9)	112 (68.7)	25 (15.3)
Rounis 2021	23	NA	NA	NA	NA	NA
Verschueren 2021	96	NA	NA	NA	NA	NA
Estevez 2020	59	NA	NA	NA	NA	NA
Hossain 2020	34	NA	NA	NA	NA	NA
Svaton 2020	64	41 (64.1)	21 (32.8)	0	2 (3.1)	0
Zhao 2019	40	NA	NA	NA	NA	NA
Jun 2020	85	NA	NA	NA	NA	NA
Lee 2020	30	NA	NA	NA	NA	NA
Mollica 2022	113	NA	NA	NA	NA	NA
IMvigor 210	263	NA	NA	NA	NA	NA
IMvigor 211	568	NA	NA	NA	NA	NA
Okuyama 2022	99	NA	NA	NA	NA	NA
Tomisaki 2022	15	NA	NA	NA	NA	NA
Bañobre 2021	54	NA	NA	NA	NA	NA
Gaucher 2021	149	NA	NA	NA	NA	NA
Afazal 2019	29	NA	NA	NA	NA	NA
Failing 2016	39	27 (69.2)	9 (23.1)	2 (5.1)	2 (5.1)	NA
Araujo 2021	114	NA	NA	NA	NA	NA
Buti 2021	104	NA	NA	NA	NA	NA
Giordan 2021	74	10 (13.5)	31 (41.9)	17 (23.0)	11 (14.9)	5 (6.8)
Husain 2021	415	NA	NA	NA	NA	NA
Peng 2021	89	42 (47.2)	21 (23.6)	9 (10.1)	1 (1.1)	1 (1.1)
Alessio 2020	491	NA	NA	NA	NA	NA
Santamaria 2019	78	NA	NA	NA	NA	NA
Ruiz 2020	135	NA	NA	NA	NA	NA
Kostine 2019	293	NA	NA	NA	NA	NA
TRIO-013/LOGiC	119	NA	NA	NA	NA	NA
Wong 2019	99	NA	NA	NA	NA	NA
Rhinehart 2018	18	NA	NA	NA	NA	NA

Sun 2016	77	NA	NA	NA	NA	NA
Wang 2017	474	NA	NA	NA	NA	NA
AXEPT	49	NA	NA	NA	NA	NA
HORIZON III	87	36 (40.9)	22 (24.9)	10 (11.4)	15 (17.0)	5 (5.7)
N016966	327	115 (35.1)	38 (11.6)	34 (10.3)	48 (14.6)	8 (2.4)
AVF2107g	159	46 (28.9)	31 (19.4)	29 (18.2)	60 (37.7)	10 (6.2)
Carrato 2013	43	22 (51.1)	12 (2.9)	6 (13.9)	3 (6.9)	0 (0)
VELOUR	113	59 (52.2)	21 (18.5)	13 (11.5)	17 (15.0)	3 (2.6)
RAISE	136	119 (46.1)	56 (21.7)	18 (6.9)	47 (18.2)	18 (6.9)
CheckMate 066	97	NA	NA	NA	NA	NA
CheckMate 067	161	NA	NA	NA	NA	NA
CheckMate 069	33	NA	NA	NA	NA	NA
Kunimitsu 2022	34	NA	NA	NA	NA	NA
Fukuokaya 2022	56	NA	NA	NA	NA	NA

Table S5. Covariates of studies reporting adjusted estimates

Study	Adjusted covariates
IMpower 130	Age, sex, race, ECOG-PS, smoking status, histology, presence of liver metastasis and PD-L1 expression
IMpower 131	Age, sex, race, ECOG-PS, smoking status, histology, presence of liver metastasis and PD-L1 expression
IMpower 150	Age, ECOG-PS, race, smoking status, histology, Teff score and EGFR mutation
POPLAR	*No adjusted estimates*
OAK	*No adjusted estimates*
Baek 2022	Age, sex, respiratory disease, viral hepatitis, antibiotics and corticosteroid use
Takada 2022	Age, sex, ECOG-PS, smoking history, mutation status (EGFR or ALK), histology, PD-L1 tumor proportion score, probiotics
Alessio 2021	*Age (<70vs ≥70 years old), gender, ECOG (0–1 vs ≥2), smoking status (current/former vs never), CNS metastases (yes vs no), bone metastases (yes vs no) and liver metastases (yes vs no).*
Balado 2021	*No adjusted estimates*
Muira 2021	Those with p-values <0.2 in the univariate analysis:<br>Histology (squamous vs non-squamous), EGFR mutation, line of chemotherapy, use of statins, NSAIDs, PPIs, opioids, and laxatives
Rounis 2021	*No adjusted estimates*
Verschueren 2021	NA
Estevez 2020	Those with p-values <0.1 in the univariate analysis: Female, use of PPI, and irAE
Hossain 2020	*No adjusted estimates*
Svaton 2020	N/A
Zhao 2019	NA
Jun 2020	Age, gender, geographic region, AFP level (>400ng/ml), BCLC (A-B vs C-D), and prior antibiotics

Lee 2020	*No adjusted estimates*
Mollica 2022	*No adjusted estimates*
IMvigor 210	Age, gender, BMI, ECOG, smoking status, histology, count of prior treatments, PD-L1 expression, serum HGB levels, count of organ sites with metastases, and presence of liver metastases.
IMvigor 211	Age, gender, BMI, ECOG, smoking status, histology, count of prior treatments, PD-L1 expression, Hb levels, count of organ sites with metastases, and presence of liver metastases.
Fukuokaya 2022	NA
Kunimitsu 2022	Age, sex, ECOG-PS, smoking status, history of operation, hemoglobin, albumin, liver metastasis and neutrophil-to-lymphocyte ratio
Okuyama 2022	Age, sex, ICI therapy treatment line, ECOG-PS at initiation of ICI therapy, tumor type, PPI use, Abs use, and exposure to radiotherapy.
Tomisaki 2022	Age, sex, ECOG-PS, hemoglobin level, liver metastasis, time from prior chemotherapy and use of antibiotics.
Bañobre 2021	*Sex (male vs female), ECOG-PS (0-1 vs ≥2), dNLR (≥3 vs <3), Metastatic sites (increment of one site), Bone metastases (yes vs no), Brain metastases (yes vs no), Liver metastases (yes vs no), Lymph node metastases (yes vs no), Peritoneal metastases (yes vs no), Antibiotic use (yes vs no), PPI use (yes vs no), Albumin (<3.5 g/dL vs ≥3.5 g/dL), Hemoglobin (<10 g/dL vs ≥10 g/dL)*
Lida 2021	N/A
Gaucher 2021	Age, gender, ECOG-PS, BMI, smoking status, alcohol consumption, a history of cardiovascular disease, and cancer duration
CheckMate 066	Age, gender, geographic region, race, ECOG-PS, metastasis stage, AJCC stage, history of brain metastasis, PD-L1 status, LDH level, BRAF mutation
CheckMate 067	Age, gender, geographic region, race, ECOG-PS, metastasis stage, AJCC stage, history of brain metastasis, PD-L1 status, LDH level, BRAF mutation
CheckMate 069	Age, gender, geographic region, race, ECOG-PS, metastasis stage, AJCC stage, history of brain metastasis, PD-L1 status, LDH level, BRAF mutation
Afzal 2019	N/A
Failing 2016	*No adjusted estimates*
Araujo 2021	N/A

Buti 2021	*No adjusted estimates*
Giordan 2021	The propensity score was estimated using multinomial logistic regression using the groups as the dependent variable and PS, gender, age, grade and the interaction between tumor location and treatment line number as independent variables.
Husain 2021	N/A
Peng 2021	*No adjusted estimates*
Alessio 2020	Age (<70 vs ≥70 years), gender (male vs female), ECOG-PS (0–1 vs ≥2), primary tumor type (NSCLC, melanoma, renal cell carcinoma and others), burden of disease (number of metastatic sites≤2 vs >2), treatment line (first vs non-first), and BMI
Santamaria 2019	Age and gender
Ruiz 2020	No adjusted estimates
Kostine 2021	N/A
TRIO013/ LOGiC	Age (≥60 years), gender, diffuse subtype, metastatic disease at presentation, and non-Asian race/ethnicity
Wong 2019	N/A
Sun 2016	Age (> 68 years), gender, stage III, and poorer ECOG PS (ECOG >/=2)
Wang 2017	N/A
AXEPT	Country, performance status, number of metastatic sites, previous use of oxaliplatin treatment, and concurrent bevacizumab treatment
HORIZON III	Age, gender, race, ECOG-PS, and serum CEA and lactate dehydrogenase (LDH) levels
N016966	Age, gender, race, ECOG-PS, and serum CEA and lactate dehydrogenase (LDH) levels
AVF2107	Age, gender, race, ECOG-PS, and serum CEA and lactate dehydrogenase (LDH) levels
Carrato 2013	Age, gender, race, ECOG-PS, and serum CEA and lactate dehydrogenase (LDH) levels
VELOUR	Age, gender, race, ECOG-PS, and serum CEA and lactate dehydrogenase (LDH) levels

RAISE	Age, gender, race, ECOG-PS, and serum CEA and lactate dehydrogenase (LDH) levels

Table S6. Summary of findings of our meta-analysis based on different cancers

Cancer	Reporting study numbers	Patients	Immune checkpoint inhibitors (PPI vs non-PPI)				Chemotherapy (PPI vs non-PPI)			
			OS	aOS	PFS	aPFS	OS	aOS	PFS	aPFS
Advanced NSCLC	17		1.36 (1.28-1.45); I^2=0%	1.32 (1.23-1.42); I^2=0%	1.36 (1.26-1.48); I^2=0%	1.51 (1.15-1.99); I^2=63%	1.12 (1.03-1.22); I^2=0%	1.04 (0.77-1.41)	1.06 (0.99-1.14); I^2=0%	0.97 (0.76-1.24)
Advanced UC	8		1.71 (1.49-1.96); I^2=0%	1.62 (1.38-1.90); I^2=0%	1.55 (1.37-1.75); I^2=0%	1.50 (1.29-1.47); I^2=22%	1.12 (0.90-1.39)	1.16 (0.92-1.46)	1.09 (0.90-1.33)	1.11 (0.89-1.38)
Melanoma	7		1.11 (0.86-1.42); I^2=62%	1.13 (0.55-2.33)	0.96 (0.75-1.23); I^2=61%	N/A	1.62 (1.09-2.41)	N/A	1.12 (0.80-1.57)	N/A
RCC	3		1.03 (0.72-1.47); I^2=16%	N/A	1.02 (0.79-1.32); I^2=0%	N/A	N/A	N/A	N/A	N/A
HCC	2		1.08 (0.82-1.42); I^2=0%	N/A	N/A	N/A	N/A	N/A	N/A	N/A
Head and neck SCC	1		1.35 (0.29-6.32)	N/A	0.44 (0.10-1.95)	N/A	N/A	N/A	N/A	N/A
CRC	11		N/A	N/A	N/A	N/A	1.20 (1.07-1.34); I^2=66%	1.17 (1.01-1.34) I^2=62%	1.17 (1.03-1.33); I^2=73%	1.16 (0.99-1.36) I^2=69%
Uncategorized cancers	10		1.56 (1.32-1.85); I^2=64%	1.33 (1.00-1.76); I^2=74%	2.39 (1.18-4.86); I^2=90%	1.52 (0.95-2.43); I^2=92%	N/A	N/A	N/A	N/A
Regimen in CRC										
FU-based agents	3						1.18 (1.00-1.40); I^2=74%	1.16 (0.93-1.46); I^2=75%	1.15 (0.97-1.36); I^2=75%	1.10 (0.88-1.39); I^2=75%
Capecitabine-based agents	3						1.02 (0.90-1.15); I^2=0%	1.01 (0.84-1.20); I^2=0%	1.07 (0.92-1.24); I^2=36%	1.20 (0.96-1.54); I^2=50%

Note: PPI, proton pump inhibitors; OS, overall survival; PFS, progression-free survival; NSCLC, non-small cell lung cancer; US, urothelial carcinoma; RCC, renal cell carcinoma; HCC, hepatocellular carcinoma; SCC, squamous cell carcinoma; CRC, colorectal carcinoma; N/A, non-available.
